# GLI3 Repressor Controls Nephron Number via Regulation of *Wnt11* and *Ret* in Ureteric Tip Cells

**DOI:** 10.1371/journal.pone.0007313

**Published:** 2009-10-07

**Authors:** Jason E. Cain, Epshita Islam, Fiona Haxho, Lin Chen, Darren Bridgewater, Erica Nieuwenhuis, Chi-Chung Hui, Norman D. Rosenblum

**Affiliations:** 1 Program in Developmental and Stem Cell Biology, The Hospital for Sick Children, Toronto Medical Discovery Towers, Toronto, Ontario, Canada; 2 Division of Nephrology, The Hospital for Sick Children, Toronto, Ontario, Canada; 3 Department of Molecular and Medical Genetics, University of Toronto, Toronto, Ontario, Canada; 4 Department of Paediatrics, University of Toronto, Toronto, Ontario, Canada; 5 Department of Laboratory Medicine and Pathobiology, University of Toronto, Toronto, Ontario, Canada; 6 Department of Physiology, University of Toronto, Toronto, Ontario, Canada; Karolinska Institutet, Sweden

## Abstract

Truncating GLI3 mutations in Pallister-Hall Syndrome with renal malformation suggests a requirement for Hedgehog signaling during renal development. HH-dependent signaling increases levels of GLI transcriptional activators and decreases processing of GLI3 to a shorter transcriptional repressor. Previously, we showed that *Shh*-deficiency interrupts early inductive events during renal development in a manner dependent on GLI3 repressor. Here we identify a novel function for GLI3 repressor in controlling nephron number. During renal morphogenesis, HH signaling activity, assayed by expression of *Ptc1-lacZ*, is localized to ureteric cells of the medulla, but is undetectable in the cortex. Targeted inactivation of *Smo*, the HH effector, in the ureteric cell lineage causes no detectable abnormality in renal morphogenesis. The functional significance of absent HH signaling activity in cortical ureteric cells was determined by targeted deletion of *Ptc1*, the SMO inhibitor, in the ureteric cell lineage. *Ptc1^−/−UB^* mice demonstrate ectopic *Ptc1-lacZ* expression in ureteric branch tips and renal hypoplasia characterized by reduced kidney size and a paucity of mature and intermediate nephrogenic structures. Ureteric tip cells are remarkable for abnormal morphology and impaired expression of *Ret* and *Wnt11*, markers of tip cell differentiation. A finding of renal hypoplasia in *Gli3* ^−/−^ mice suggests a pathogenic role for reduced GLI3 repressor in the *Ptc1^−/−UB^* mice. Indeed, constitutive expression of GLI3 repressor via the *Gli3^Δ699^* allele in *Ptc1^−/−UB^* mice restores the normal pattern of HH signaling, and expression of *Ret* and *Wnt11* and rescued the renal phenotype. Thus, GLI3 repressor controls nephron number by regulating ureteric tip cell expression of *Wnt11* and *Ret*.

## Introduction

Development of the permanent mammalian kidney (the metanephros) is dependent on growth and branching of the ureteric bud and its daughter branches, a process termed renal branching morphogenesis. At the onset of this process, the ureteric bud elongates towards and invades the metanephric mesenchyme before undergoing spatial specification into ‘ureteric stalk’ and ‘ureteric tip’ domains. Reciprocal inductive interactions between the ureteric tip and surrounding metanephric mesenchyme results in division of the ureteric tip, forming the first of a series of ureteric branches, which ultimately constitute the mature collecting duct system. Simultaneously, each ureteric bud tip induces adjacent metanephric mesenchyme cells to undergo a mesenchyme-epithelial transformation and form the epithelial components extending from the glomerulus to the distal tubule, a process known as nephrogenesis. The number of nephrons formed is directly related to the number of ureteric branches and their inductive capacity. Severe reductions in nephron number, characteristic of renal hypoplasia/dysplasia, are the leading cause of childhood renal failure. More subtle defects in nephron number have been associated with the development of adult-onset essential hypertension and chronic renal failure [1], [2], [3], [4], [5].

The Hedgehog-GLI signaling pathway plays critical roles during mammalian kidney development. GLI proteins function downstream of Hedgehog's (HH), extracellular proteins, and Patched (PTC) and Smoothened (SMO), cell surface proteins. HH ligand signals upon binding to its receptor, PTC, relieving PTC-mediated inhibition of SMO, a transmembrane protein. In this state, SMO interacts with a molecular complex including Costal-2 (Cos2), Fused (Fu) and Suppressor of Fused (SuFu), ultimately resulting in translocation of GLI protein family members into the nucleus where they act as transcriptional activators. In the absence of HH ligand, PTC inhibits SMO and prevents its interaction with the Cos2-Fu-SuFu complex resulting in C-terminal cleavage of GLI protein, which translocates to the nucleus and acts as a transcriptional repressor. In vertebrates, three GLI family members, GLI1, GLI2 and GLI3, mediate HH signals. During murine embryogenesis, GLI1 and GLI2 are believed to function primarily as transcriptional activators while GLI3 is believed to function primarily as a transcriptional repressor [6], [7].

The balance of GLI activator and repressor activities is critical during renal morphogenesis. Mutations that are predicted to generate a truncated protein similar in size to GLI3 repressor are observed in humans with Pallister-Hall Syndrome (PHS) and renal dysplasia [8]. The pathogenic role of constitutive GLI3 repressor activity during renal morphogenesis is further demonstrated by the renal dysplastic phenotype in mice engineered to express GLI3 repressor in a dominant manner [9] and in S*hh*-deficient mice [10]. Dysplastic kidney tissue in *Shh*-deficient mice is characterized by sustained GLI3 repressor expression in the face of decreased levels of GLI activators (GLI1, GLI2 and full-length GLI3), resulting in a shift in the balance of GLI activators and GLI repressors in favor of repressor [10]. Remarkably, genetic elimination of *Gli3* in the *Shh* null background restores expression of GLI activators and normalizes renal morphogenesis [10]. The expression of *Shh* in ureteric cells suggests that it may control renal development via direct effects in the ureteric cell lineage. While conditional inactivation of *Shh* in ureteric cells results in renal hypoplasia, characterized by reduced kidney size and glomerular number [11], the dependency of this pathogenic phenotype on *Shh* signaling in ureteric cells is unknown.

Here we define the specific function of HH signaling in the ureteric cell lineage during murine kidney development, in genetic models of deficient or constitutively active signaling. HH signaling activity is specifically restricted to the ureteric cells of the medulla and ureter but is absent from the ureteric cell tips of the renal cortex. Genetic inactivation of *Smo* in the ureteric cell lineage exerted no deleterious effects on renal morphogenesis. In contrast, genetic inactivation of *Ptc1* in the ureteric cell lineage caused ectopic HH signaling activity in ureteric tip cells, impaired ureteric tip cell-specific gene expression and renal hypoplasia. Genetic inactivation of *Gli3* alone, the primary GLI repressor, resulted in a similar phenotype suggesting a critical role for GLI3 repressor. Indeed, introduction of a constitutively active GLI3 repressor in a *Ptc1*-deficienct background normalized the renal phenotype, restored the normal domain of HH signaling activity and rescued expression of genes specific to ureteric tip cells and required for their functions. We propose a model in which SHH-SMO signaling controls the spatial generation of GLI3 repressor, which is required in the cortical ureteric cells for ureteric tip cell-specific gene expression and cell function.

## Results

### Spatial Restriction of HH Signaling Activity to the Renal Medulla and Ureter

**Figure 1:**
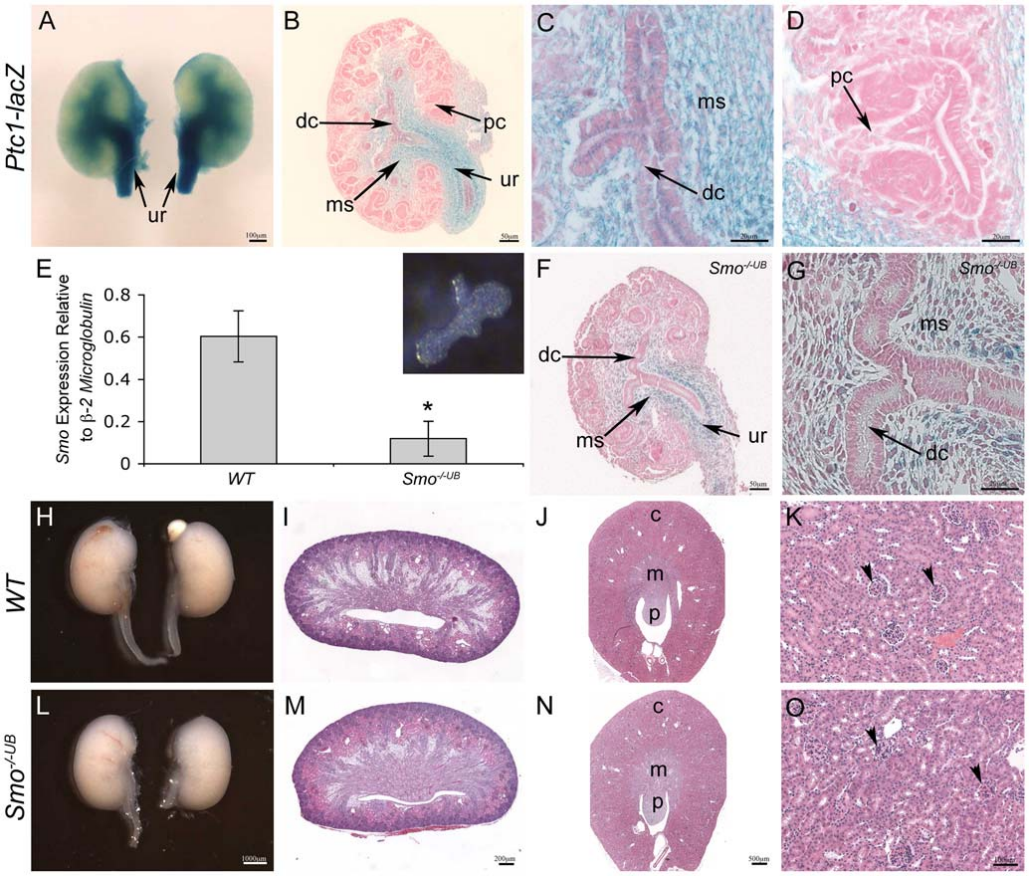
*Ptc1-lacZ* expression and site of HH signaling activity in the developing murine kidney. (A–D) Whole mount X-gal staining of kidney tissue isolated from E13.5 *Ptc1-lacZ* reporter mice reveals strong localization of HH signaling activity to the cells surrounding the presumptive ureter (ur), medullary stroma (ms) and weakly to the epithelium of the presumptive ureter and distal collecting ducts (dc). (D) *Ptc1-lacZ* is not observed in any structures of the presumptive renal cortex. (E) Quantitative real-time PCR of isolated E11.5 ureteric buds (inset) demonstrates decreased *Smo* mRNA transcripts in *Smo^−/−UB^* kidneys compared to *WT* littermates (*WT* vs. *Smo^−/−UB^*: 0.60±0.12 vs. 0.12±0.08, p<0.05). (F,G) *Ptc1-lacZ* expression in *Smo^−/−UB^* kidneys at E13.5. *Ptc1-lacZ* expression is maintained in the cells surrounding the ureter and medullary stroma but is markedly reduced in the ureteric cells of the ureter and distal collecting ducts. (H,I,L,M) Macroscopic and histological analysis of newborn *Smo^−/−UB^* kidneys is comparable to *WT* littermates. (J,K,N,O) Analysis of mature *Smo*-deficient kidneys at PN30 demonstrates no histological abnormalities. c  =  cortex, m  =  medulla, p  =  papilla, pc  =  proximal collecting duct, arrowhead  =  mature glomerulus.

To begin to further investigate the role of SHH signaling during renal morphogenesis, we examined the expression of *Ptc1* utilizing the *Ptc1-lacZ* reporter mouse [12]. Since *Ptc1* is a downstream target of HH signaling, *Ptc1-lacZ* expression is indicative of the site of HH signaling activity [12], [13], [14]. In the *WT* (*Ptc1^lacZ/+^*) kidney at E13.5, *Ptc1-lacZ* is strongly localized to cells surrounding the presumptive ureter and the presumptive medullary stroma (Figure 1A,B), consistent with the pattern of *Ptc1* mRNA expression [11]. *Ptc1-lacZ* is also weakly localized to the epithelium of the presumptive ureter and the distal or medullary collecting ducts (Figure 1A–C). Interestingly, *Ptc1-lacZ* expression is not observed in any structures of the presumptive renal cortex, suggesting that HH signaling activity is restricted to the ureter and medullary regions of the developing kidney (Figure 1B,D). At a later stage (E18.5) of kidney development, a similar pattern of expression is maintained in the cells surrounding the ureter and medullary stroma (Figure S1A–C). However, at E18.5, *Ptc1-lacZ* expression is not observed in any epithelial structures (Figure S1A–C). Taken together, *Ptc1-lacZ* expression in both ureteric and metanephric mesenchyme-derived structures suggests a role for SHH function in both the ureteric bud and metanephric mesenchyme lineages of the early developing kidney but only in the presumptive ureter and medullary regions.

### SHH-SMO-Dependent Signaling is not Required in the Ureteric Cell Lineage

We began to investigate the possible autocrine functions of SHH-SMO-dependent signaling by generating a loss of function model for HH signaling in the ureteric cell lineage. *Smoothened* is required for the transduction of all HH signals. Similar to *Shh* inactivation, inhibition of SMO with a steroidal alkaloid, cyclopamine, results in sustained GLI3 repressor in the absence of GLI activators [10]. Homozygous germline deficiency of *Smo* in the mouse results in embryonic lethality prior to the commencement of metanephric development [15]. Therefore, we utilized *Hoxb7creEGFP* mice to generate a murine model in which *Smo* is genetically inactivated in the ureteric cell lineage [16], [17], thereby eliminating SHH-SMO-dependant signaling.

Targeted deficiency of *Smo* in the ureteric cell lineage did not adversely effect survival since mutants were recovered in the near expected Mendelian ratios (Table S1). To confirm that *Smo* is abolished in *Smo^−/−UB^* mutants, we performed quantitative real-time PCR using ureteric bud tissue isolated at E11.5, a stage that closely follows metanephric induction. Interestingly, *Smo* mRNA transcripts were decreased by ∼80% in *Smo^−/−UB^* ureteric cells compared to *WT* (*WT* vs. *Smo^−/−UB^*: 0.60±0.12 vs. 0.12±0.08, p<0.05) (Figure 1E). Consistent with a loss of *Smo*, analyses of *Ptc1-lacZ* in *Smo^−/−UB^* kidneys at E13.5 revealed a marked reduction in HH signaling activity in the ureteric cells of the ureter and medullary collecting ducts (Figure 1F,G). Examination of the gross anatomical and histological features of newborn *Smo^−/−UB^* kidneys revealed no major differences compared to *WT* kidneys (Figure 1H,I,L,M). Consistent with this observation, a more detailed analysis of nephrogenic and ureteric structures using immunofluorescence microscopy and RNA *in situ* hybridization revealed that glomerulogenesis, nephrogenesis, nephron segmentation, ureteric branching morphogenesis and ureteric tip cell-specific gene expression was normal in *Smo^−/−UB^* kidneys (Figure S2). In order to determine if HH-SMO-dependant signaling is required in the mature kidney, we also examined *Smo^−/−UB^* kidneys at PN30. No histological abnormalities were detected in the *Smo^−/−UB^* kidneys (Figure 1J,K,N,O). Taken together, these results demonstrate that HH-SMO-dependent signaling is not required in the ureteric cell lineage and suggest that SHH has no autocrine function [in the ureteric cells] during renal morphogenesis.

### A Model of Cortical HH Signaling Activity in the Embryonic Kidney

Our results demonstrate that HH signaling activity is absent from the renal cortex. We determined the importance of this spatial restriction of HH signaling activity by generating a gain-of-function model in which HH signaling is ectopically activated in the cortical ureteric epithelium.

**Figure 2:**
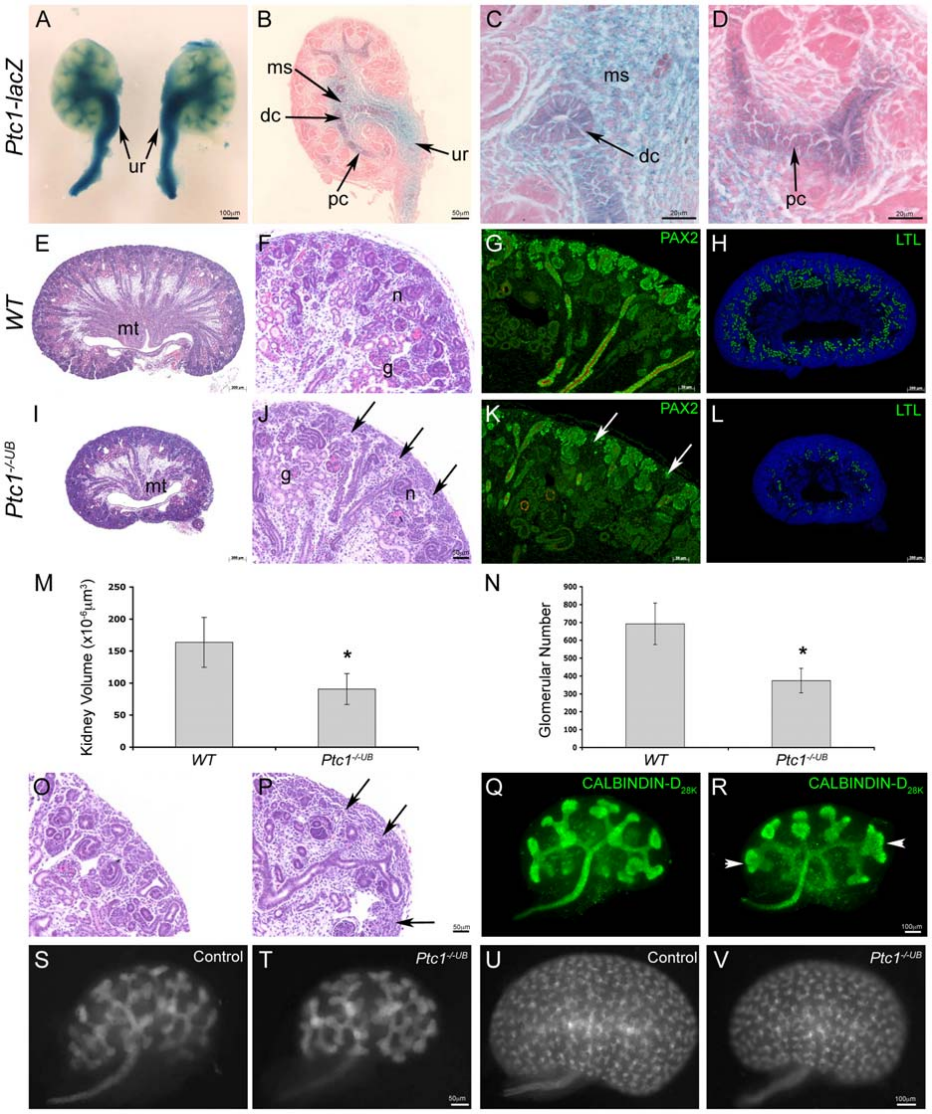
Renal hypoplasia in mice with *Ptc1*-deficiency in the ureteric cell lineage. (A–D) In *Ptc1* mutant kidneys (*Ptc1^−/−^* ^UB^), Ptc1*-lacZ* expression is maintained in the cells surrounding the presumptive ureter (ur) and medullary stroma (ms). (A–C) HH signaling activity is upregulated in the epithelium of the presumptive ureter and distal collecting ducts (dc). (D) Ectopic HH signaling activity is observed in the proximal collecting ducts (pc) and weakly in ureteric bud tips. (E–L) Histological and immunofluorescence analysis of *WT* and *Ptc1^−/−UB^* kidneys at E18.5. *Ptc1*-deficient mice exhibit small kidneys, reduced density of medullary epithelial tubules (mt) and a paucity of mature glomeruli (g) and nephrogenic intermediate structures (n) and sparsity of the nephrogenic zone (arrows). (G,K) PAX2 (green) positive nephrogenic structures are reduced in the *Ptc1^−/−UB^* kidneys. (H,L) LTL (green) positive proximal tubules are markedly reduced in *Ptc1^−/−UB^* kidneys. (M) Kidney volume in *Ptc1^−/−UB^* mutants is reduced by 45% in comparison to *WT* littermates (p<0.01). (N) *Ptc1^−/−UB^* kidneys demonstrate a 45% reduction in mature glomerular number compared to *WT* littermates (p<0.01). (O,P) Histological analysis also revealed a sparsity of the nephrogenic zone (arrows) and reduction in the number of nephrogenic structures in *Ptc1^−/−UB^* compared to *WT* kidneys at E15.5. (Q,R) Analysis of ureteric branching morphogenesis in *WT* and *Ptc1^−/−UB^* kidneys at E12.5. The number of ureteric branches was comparable between *WT* and *Ptc1^−/−UB^* kidneys. However, *Ptc1^−/−UB^* kidneys exhibited abnormal ureteric tip morphology (arrowhead). (S–V) Whole mount GFP immunofluorescence of control and mutant kidneys. Kidney size and branching is mildly reduced at E13.5 (S,T) and is more pronounced at E15.5 (U,V).

*Patched1* is a negative regulator of the HH signaling pathway. In the absence of *Ptc1*, repression of HH target genes is alleviated, even in the absence of SHH ligand. Homozygous germline deficiency of *Ptc1* in the mouse results in embryonic lethality prior to the commencement of metanephric development [12]. Therefore, we utilized *Hoxb7creEGFP* mice to generate a murine model in which *Ptc1* is genetically inactivated in the ureteric cell lineage [16], [17] (refer to Methods). To confirm that inactivation of *Ptc1* results in a constitutively active HH signaling pathway in the ureteric cell lineage, we analyzed *Ptc1-lacZ* expression in *Ptc1^−/−UB^* kidneys. While *Ptc1-lacZ* expression is maintained in the cells surrounding the presumptive ureter and presumptive medullary stroma (Figure 2A,B), *Ptc1-lacZ* is markedly upregulated in the epithelium of the presumptive ureter and distal collecting ducts in *Ptc1^−/−UB^* kidneys (Figure 1C vs. Figure 2C). Remarkably, *Ptc1-lacZ* is ectopically expressed throughout the cortical or proximal collecting ducts and in the ureteric bud tips (Figure 2A,B,D), albeit in a mosaic pattern (Figure S1F). A similar pattern of upregulated and ectopic ureteric bud epithelial *Ptc1-lacZ* expression was also observed at a later stage (E18.5) of kidney development (Figure S1D-F). To confirm that *Ptc1* inactivation occurs by an early stage in renal morphogenesis, we assayed expression of *Ptc1* mRNA, a surrogate marker of HH signaling activity, in ureteric bud tissue isolated from E11.5 kidney. Quantitative real-time PCR using primers designed for an undisrupted region of the mutant *Ptc1* transcript demonstrated a 50-fold increase in *Ptc1* mRNA transcripts in *Ptc1^−/−UB^* compared to *WT* ureteric cells (*WT* vs. *Ptc1^−/−UB^*: 2.22±0.02 vs. 108.51±6.47, p<0.001) (Figure S3H). Together, these results show that genetic elimination of PTC1 in the ureteric cell lineage leads to increased and ectopic HH signaling activity in the developing kidney.

### Ectopic HH Signaling Activity in the Proximal Epithelium Causes Renal Hypoplasia

Viable neonatal PTC1-deficient pups could not be recovered (Table S2). However, live *Ptc1^−/−UB^* mutant embryos were recovered in expected Mendelian ratios at all embryonic time points analyzed (Table S2). Macroscopic analyses of *Ptc1^−/−UB^* embryos revealed severe exencephaly, with 100% penetrance. Otherwise, *Ptc1^−/−UB^* embryos were indistinguishable from *WT* littermates (Figure S3). It is therefore likely that *Ptc1^−/−UB^* embryos reach term and die and/or are cannibalized immediately following birth.

Analysis of *Ptc1^−/−UB^* kidneys at E18.5 revealed renal hypoplasia, characterized by a 45% reduction in kidney volume (*WT* vs. *Ptc1^−/−UB^*: 163.67±38.87×10^−6^ µm^3^ vs. 90.72±24.05×10^−6^ µm^3^, p<0.01) and reduced density of medullary epithelial tubules (Figure 2E,I,M). The extent of reduction in *Ptc1^−/−UB^* kidney size was variable, exhibiting a 1.7 fold range, but was 100% penetrant. Histological analyses of the renal cortex of *Ptc1^−/−UB^* kidneys revealed a paucity of mature glomeruli and nephrogenic intermediate structures in the nephrogenic zone (Figure 2F,J). Glomerular number in *Ptc1^−/−UB^* kidneys was decreased by 45% in comparison to *WT* littermates (*WT* vs. *Ptc1^−/−UB^*: 692.22±116.35 vs. 374.25±68.79, p<0.001) (Figure 2N). A deficiency in nephrogenic structures in *Ptc1^−/−UB^* kidneys was confirmed by examining the expression of PAX2, a marker of condensing mesenchyme and early nephrogenic structures (Figure 2G,K) and LTL, a marker of proximal tubules (Figure 2 H,L). A reduction in the number of intermediate nephrogenic structures was also observed in *Ptc1^−/−UB^* kidneys by histology at E15.5 (Figure 2O,P). However, no difference in renal histology or the number of nephrogenic intermediate structures was observed between *WT* and *Ptc1^−/−UB^* kidneys at E13.5 suggesting that nephrogenesis is unaffected at that stage (Figure S4). Together, these results demonstrate that PTC1-deficiency leads to deficient nephrogenesis.

Ureteric branching is required for nephron formation. Since ureteric branch tips induce contiguous metanephric mesenchyme cells to engage in nephron formation, the number of ureteric branches is considered to be a critical determinant of the number of nephrons generated. To determine the effect of *Ptc1*-deficiency on early ureteric branching morphogenesis we quantified ureteric branching at E12.5 by whole mount immunofluorescence using Calbindin-D_28K_, a marker of ureteric bud epithelium. No significant difference in the number of ureteric branches was observed between *WT* and *Ptc1^−/−UB^* kidneys (Figure 2Q,R and Figure S4F). However, we did observe abnormalities in the ureteric branch pattern. In contrast to *WT* kidneys, several irregularly shaped and dilated ureteric bud tips were observed in *Ptc1^−/−UB^* kidneys (Figure 2Q,R). Analyses of ureteric branching morphogenesis a day later at E13.5 revealed a mild reduction in ureteric branch number in *Ptc1^−/−UB^* kidneys compared to controls (Figure 2S,T). At E15.5, this reduction was more even more pronounced (Figure 2U,V). Importantly, normal renal architecture is maintained in *Ptc1^−/−UB^* kidneys. Taken together, these results demonstrate that ectopic HH signaling activity in proximal ureteric cells causes renal hypoplasia, characterized by marked defects in the formation of nephrogenic structures, reduced ureteric branching and a qualitative defect in early stage ureteric branch tips.

### Effect of Ptc1-Deficiency on Proliferation and Apoptosis

**Figure 3:**
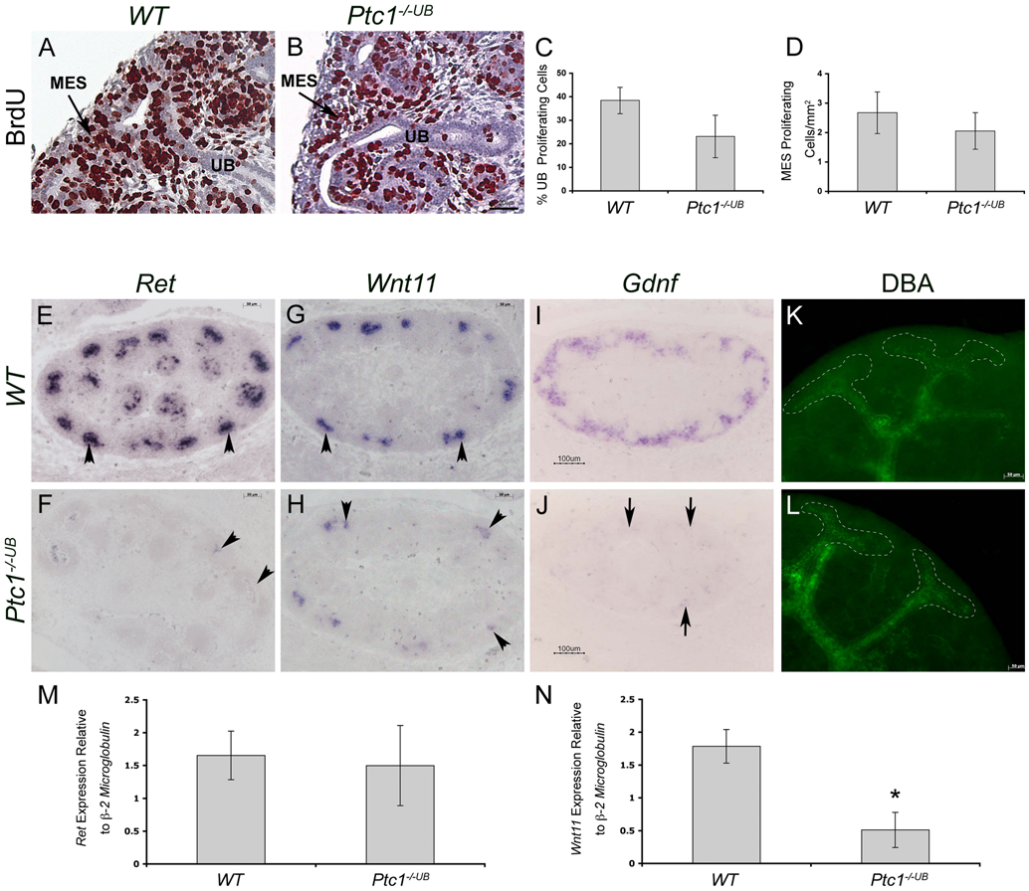
Ectopic HH signaling activity in the proximal epithelium impairs ureteric tip cell–specific gene expression. (A–D) Analysis of cell proliferation in E13.5 kidney tissue using in situ BrdU incorporation assay. BrdU (brown color) is decreased in ureteric cells in *Ptc1^−/−UB^* kidney compared to *WT*. (C) Quantitative analysis of ureteric cell proliferation. Ureteric cell proliferation, quantitated as the percent of BrdU-positive ureteric cells, was decreased in *Ptc1*-deficient kidneys (p = 0.05). (D) Quantitative analysis of mesenchymal cell proliferation. Mesenchymal cell proliferation, quantitated as the number of BrdU positive cells per mm^2^ of renal tissue was comparable in *WT* and *Ptc1^−/−UB^* kidneys. ub = ureteric bud, mes = metanephric mesenchyme. (E–H) RNA *in situ* hybridization demonstrates expression of *Ret* and *Wnt11* exclusively to the ureteric bud tips (arrowhead) in *WT* kidney tissue. In contrast, these mRNA transcripts are either absent or markedly reduced in *Ptc1^−/−UB^* kidney tissue (arrowhead). (I,J) RNA *in situ* hybridization demonstrates that *Gdnf* is markedly reduced in metanephric mesenchyme of *Ptc1^−/−UB^* kidneys (arrows). (K,L) DBA-lectin localizes predominantly to the ureteric stalk in *WT* kidneys and is excluded from the ureteric tips at E13.5. In *Ptc1^−/−UB^* kidneys DBA-lectin is observed throughout the ureteric tips and ureteric stalks. (M,N) Quantitative real-time PCR of isolated *WT* and *Ptc1^−/−UB^* ureteric buds at E11.5. (M) *Ret* expression is similar between *WT* and *Ptc1^−/−UB^* ureteric cells. (N) *Wnt11* expression is significantly decreased in *Ptc1^−/−UB^* ureteric cells (p<0.05).

Cell proliferation is crucial for ureteric branching morphogenesis and nephrogenesis. Since SHH signaling controls renal cell proliferation [10] we investigated the possible contributions of abnormal cell proliferation to the hypoplastic phenotype in PTC1-deficient kidneys at E13.5, a time point prior to the onset of the phenotype. Analysis of cell proliferation, using an *in situ* BrdU incorporation assay, revealed a 40% decrease in ureteric bud cell proliferation in *Ptc1^−/−UB^* kidneys at E13.5 (*WT* vs. *Ptc1^−/−UB^*: 38.4±5.6 vs. 23.1±9.0, p = 0.05) (Figure 3A–C). In contrast, no significant difference in mesenchymal cell proliferation was detected (*WT* vs. *Ptc1^−/−UB^*: 2.68±0.7 vs. 2.05±0.6, p = 0.25) (Figure 3A,B,D).

Mesenchyme cell survival is critical to nephrogenesis. Moreover, elevated ureteric bud apoptosis has been implicated in the pathogenesis of renal hypoplasia [18]. Accordingly, we determined whether cell survival was impaired in PTC1-deficient kidneys at E13.5 (Figure S5). Analysis of apoptosis, using a TUNEL assay, revealed no significant difference in ureteric bud or metanephric mesenchyme cell death in *Ptc1^−/−UB^* kidneys.

Taken together, these results suggest that enhanced and ectopic HH signaling activity in the ureteric cell lineage decreases ureteric cell proliferation but does not affect apoptosis.

### Ectopic HH Signaling Activity in the Proximal Ureteric Epithelium Impairs Expression of *Wnt11* and *Ret* in Ureteric Bud Tip Cells

Our findings of renal hypoplasia, abnormal ureteric tip morphology and decreased ureteric cell proliferation (in *Ptc1^−/−UB^* kidneys) suggested that ectopic HH signaling activity in ureteric tips disrupts normal ureteric tip function.

Specification of ureteric bud tip cells distinct from ureteric stalk cells is essential for ureteric branching morphogenesis and nephrogenesis. Determination of ureteric tip cell fate is dependant on *Gdnf*/*Ret* signaling [19]. Furthermore, *Gdnf*/*Ret* signaling is required for the maintenance of *Wnt11* expression, also in the ureteric bud tips cells [20], [21]. Conversely, *Wnt11* promotes *Gdnf* expression in the surrounding metanephric mesenchyme suggesting that *Gdnf*, *Ret* and *Wnt11* participate in an autoregulatory feedback loop to regulate ureteric branching morphogenesis [20]. We examined the effect of ectopic HH signaling activity on ureteric tip cell-specific gene expression using *in situ* hybridization. In contrast to *WT* kidneys, expression of *Ret* and *Wnt11* was markedly reduced in the majority of ureteric bud tips in *Ptc1^−/−UB^* kidneys at E13.5 (Figure 3E–H). Consistent with the autoregulatory feedback loop, expression of *Gdnf* in the surrounding metanephric mesenchyme was also markedly reduced in the *Ptc1^−/−UB^* kidneys (Figure 3I,J). The specificity of decreased *Gdnf* expression in *Ptc1^−/−UB^* kidneys is demonstrated by normal expression of *Osr1*, *Six2*, and CITED1, each of which marks mesenchymal precursor cells [22], [23], [24], [25], [26]; *Wnt4*, a marker of pretubular aggregates [27]; and *Wnt9b* which is required for the earliest inductive response in metanephric mesenchyme and acts upstream of *Wnt4* [28] (Figure S6). To further investigate the ontogeny of abnormal ureteric tip cell gene expression in *Ptc1^−/−UB^* mice, we assayed *Ret* and *Wnt11* expression in isolated ureteric bud tissue using quantitative real-time PCR. At E11.5, a stage that immediately follows induction of the metanephric mesenchyme by the ureteric bud, expression of *Ret* was comparable between *WT* and *Ptc1^−/−UB^* ureteric cells (*WT* vs. *Ptc1^−/−UB^*: 1.65±0.37 vs. 1.50±0.61, p = 0.78) (Figure 3M). In contrast, *Wnt11* expression was reduced by ∼70% in *Ptc1^−/−UB^* ureteric cells (*WT* vs. *Ptc1^−/−UB^*: 1.79±0.26 vs. 0.51±0.27, p<0.05) (Figure 3N). Taken together, these results demonstrate that ectopic HH signaling activity in the proximal ureteric epithelium specifically impairs the expression of *Wnt11* and *Ret* in ureteric tip cells.

Our results indicate that HH signaling activity is normally restricted to the distal ureteric cells of the ureter and medulla. Given the impairment of tip cell gene expression, we investigated the possibility that HH signaling activity biases ureteric cells towards a distal cell fate. To determine whether *Ptc1^−/−UB^* tips cells had adopted characteristics of the ureteric stalk we performed DBA-lectin whole mount immunofluorescence microscopy on *WT* and *Ptc1^−/−UB^* kidneys at E13.5. DBA is a marker of the ureteric stalk but not the ureteric tip [29]. DBA expression was observed in ureteric tips in *Ptc1^−/−UB^* kidneys in a mosaic pattern but rarely in *WT* kidneys (Figure 3K,L and Figure S5E–H). The expression of *Wnt7b*, another marker of ureteric stalk that is absent from the ureteric tips [30], was comparable between *WT* and *Ptc1^−/−UB^* kidneys. Since ectopic HH signaling activity is also observed in proximal collecting ducts in mutant kidneys, we next investigated the possibility that HH signaling activity biases cortical collecting ducts towards a more medullary/distal cell fate. Expression of uroplakin III, a marker of the transitional epithelium of the ureter and renal pelvis, was unchanged in the *Ptc1^−/−UB^* kidneys (Figure S7A–F). Similarly, the localization of αSMA, a marker of the smooth muscle population surrounding the ureter, was also unaltered (Figure S7G–L). Together, these results suggest that ectopic HH signaling activity in the proximal epithelium does not induce a distal ureteric bud cell fate. Furthermore, increased HH signaling activity in the distal epithelium (ureter and distal collecting ducts) has no deleterious effects on these structures.

### Reduced Levels of GLI Repressor in the Renal Cortex Result in Renal Hypoplasia

The spatial restriction of HH signaling activity from the renal cortex during normal renal morphogenesis suggests that the cortex is a GLI repressor-dominant domain. We hypothesized that the deleterious effects of ectopic HH signaling in the proximal ureteric cells in *Ptc1^−/−UB^* kidneys is due to a reduction in local GLI repressor levels. We addressed this hypothesis by analyzing kidneys in *Gli3*-deficient embryos.

**Figure 4:**
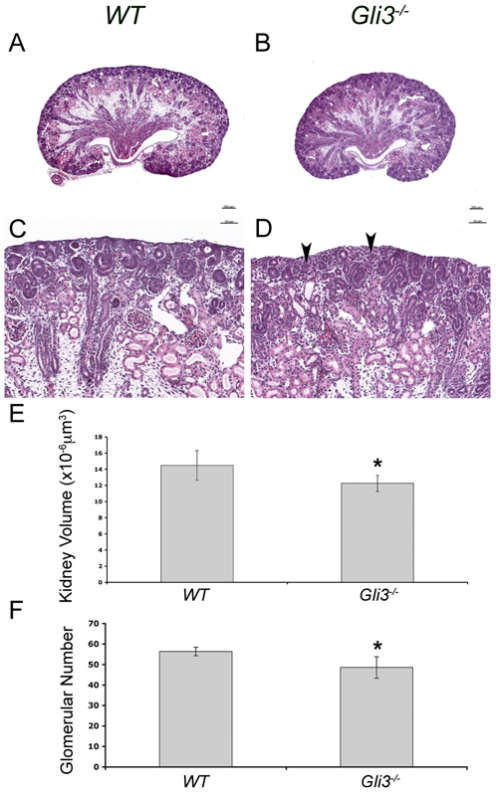
Loss of GLI repressor results in renal hypoplasia. (A–F) Analysis of *Gli3^−/−^* kidneys E18.5. Histological analysis of *Gli3* deficient kidneys revealed a reduction in kidney size (A,B) and a reduced density of the nephrogenic zone (C,D). (E) Kidney volume was reduced by 15% in *Gli3*-deficient kidneys (p<0.05). (F) The number of mature glomeruli was reduced by 15% in *Gli3*-deficient kidneys (p<0.05).

GLI3 is the primary source of GLI repressor in mammalian cells [31]. Mice with homozygous deficiency in *Gli3* die soon after birth or in late gestation [32]. We were able to recover viable *Gli3*-deficient embryos as late as E18.5. *Gli3*-deficient embryos exhibited polysyndactyly and occasional exencephaly but were similar in size to *WT* littermates (data not shown). *Gli3^+/XtJ^* mice kidneys were indistinguishable from *WT* littermates (data not shown). Analysis of *Gli3^XtJ/XtJ^* kidneys at E18.5 revealed mild hypoplasia, characterized by a 15% decrease in kidney volume (p<0.05) and 15% reduction in glomerular number (p<0.05) compared to *WT* littermates (Figure 4). Otherwise, glomerulogenesis, nephron segmentation, and smooth muscle and urothelium differentiation was normal in *Gli3^XtJ/XtJ^* kidneys (Figure S8). These results are consistent with a functional role for GLI repressor in the renal cortex during renal morphogenesis.

### GLI3 Repressor is Required for Ureteric Tip Cell Expression of *Wnt11* and *Ret*

**Figure 5:**
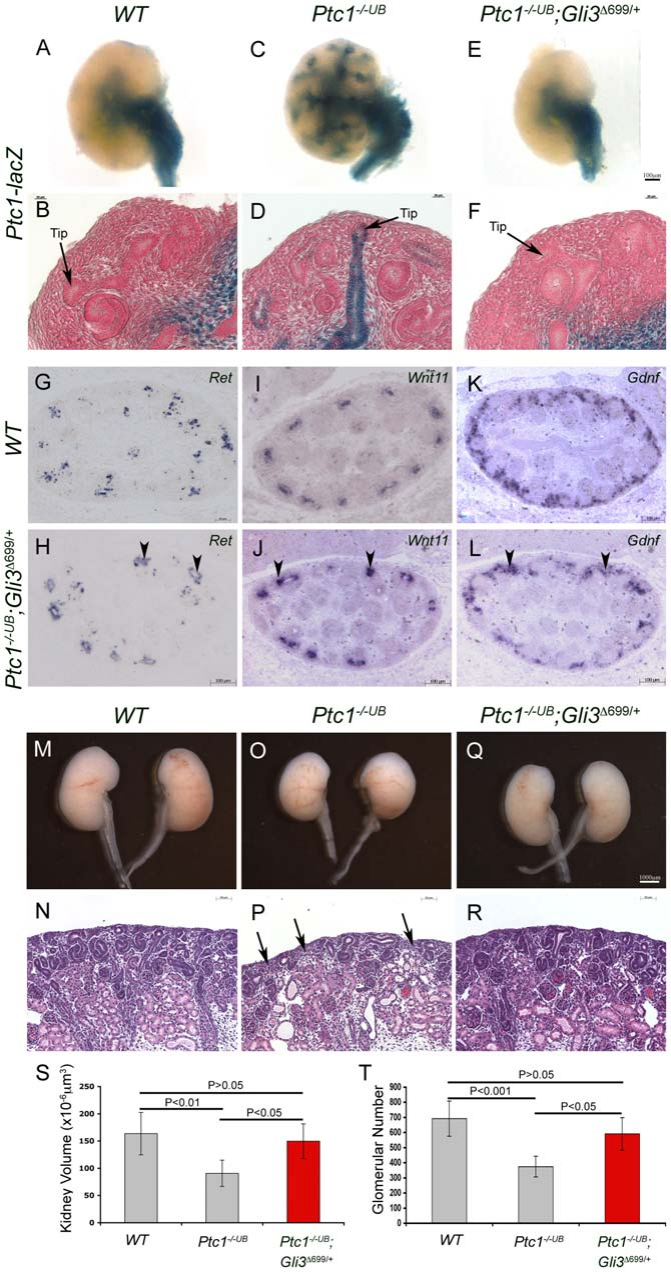
GLI3 repressor is required for ureteric tip cell-specific gene expression and function. (A–F) Normal of HH signaling activity is restored in *Ptc1^−/−UB^;Gli3^Δ699/+^* kidneys at E13.5. In contrast to *Ptc1^−/−UB^* kidneys, *Ptc1-lacZ* expression is absent from the cortical collecting ducts and ureteric bud tips in *Ptc1^−/−UB^;Gli3^Δ699/+^* kidneys. (G–L) Expression of *Ret*, *Wnt11* and *Gdnf* was restored in *Ptc1^−/−UB^;Gli3^Δ699/+^* kidneys at E13.5. Analysis of *Ptc1^−/−UB^;Gli3^Δ699/+^* kidneys at E18.5 revealed a restoration in kidney size (M,O,Q) and density of the nephrogenic zone (N,P,R). Kidney volume (S) and glomerular number (T) is rescued in *Ptc1^−/−UB^;Gli3^Δ699/+^* kidneys to comparable levels to *WT* littermates.

We further tested the contribution of GLI repressor in the proximal epithelium by reinstating GLI3 repressor levels in the *Ptc1^−/−UB^* mutants. We generated mice with both *Ptc1*-deficiency targeted to the ureteric cells and with one allele of *WT Gli3* replaced with *Gli3^Δ699^*, a constitutively active repressor form of GLI3. To determine what effect, if any, the reinstatement of GLI3 repressor had on HH signaling activity we analyzed *Ptc1-lacZ* in the *Ptc1^−/−UB^;Gli3^Δ699/+^* kidneys. In the *Ptc1^−/−UB^;Gli3^Δ699/+^* kidney at E13.5, *Ptc1-lacZ* expression is maintained in the cells surrounding the presumptive ureter and medullary stroma, consistent with the expression in *WT* and *Ptc1^−/−UB^* kidneys (Figure 5A,C,E). However, in contrast to ectopic *Ptc1-lacZ* expression throughout the proximal ureteric cells in *Ptc1^−/−UB^* kidneys, the proximal collecting ducts and ureteric cell tips are devoid of *Ptc1-lacZ* expression in *Ptc1^−/−UB^;Gli3^Δ699/+^* kidneys (Figure 5A–F). These results suggest that reinstatement of GLI3 repressor in *Ptc1^−/−UB^* kidneys, restores the normal pattern of HH signaling activity to the medullary and ureter domains and eliminates ectopic HH signaling activity in the renal cortex. Next we analyzed ureteric tip cell gene expression in *Ptc1^−/−UB^;Gli3^Δ699/+^* kidneys. Remarkably, expression of *Ret* and *Wnt11* was restored to comparable levels of that observed in *WT* and *Gli3^Δ699/+^* controls suggesting a rescue of ureteric tip cell differentiation (Figure 3E,H, Figure 5G,J, Figure S9). Furthermore, consistent with a rescue of *Ret* and *Wnt11* expression, *Gdnf* expression was also restored by the reinstatement of GLI3 repressor (Figure 3I,J, Figure 5K,L, Figure S9). Moreover, macroscopic analysis of *Ptc1^−/−UB^;Gli3^Δ699/+^* kidneys at E18.5 demonstrated a rescue in kidney size, comparable to that observed in *WT* littermates (Figure 5M,O,Q). In fact, kidney volume was significantly restored from 55% in *Ptc1^−/−UB^* kidneys alone, to 92% in *Ptc1^−/−UB^;Gli3^Δ699/+^* kidneys in comparison to *WT* kidney volume (*WT* vs. *Ptc1^−/−UB^;Gli3^Δ699/+^*, p>0.05; *Ptc1^−/−UB^* vs. *Ptc1^−/−UB^;Gli3^Δ699/+^*, p<0.05) (Figure 5S). Remarkably, histological analyses of the renal cortex also revealed a rescue in nephrogenesis in *Ptc1^−/−UB^;Gli3^Δ699/+^* kidneys (Figure 5N,P,R). In contrast to a 45% deficit in *Ptc1^−/−UB^* kidneys, glomerular number in *Ptc1^−/−UB^;Gli3^Δ699/+^* kidneys was significantly restored to 85% of that of *WT* littermates (*WT* vs. *Ptc1^−/−UB^;Gli3^Δ699/+^*, p>0.05; *Ptc1^−/−UB^* vs. *Ptc1^−/−UB^;Gli3^Δ699/+^*, p<0.05) (Figure 5T). Taken together, these results demonstrate a requirement for GLI3 repressor for ureteric tip cell gene expression and function.

## Discussion

Disruption of renal development in humans with Pallister-Hall Syndrome and truncating GLI3 mutations [33] and mice with elevated levels of GLI3 repressor [9], [10] provides compelling evidence in favor of a critical role for GLI3-dependent signaling during mesenchymal-epithelial interactions during early stages of metanephric development. However, the functions of HH signaling during subsequent morphogenic events including nephrogenesis are unknown.

Here, we demonstrate that domains of GLI-dependent activator and repressor function are spatially patterned during renal morphogenesis. We investigated the functional significance of these domains in the ureteric cell lineage using genetic murine models of deficient or constitutively active HH signaling. *Smo*-deficiency targeted to the ureteric cell lineage does not disrupt kidney development, demonstrating HH-dependent GLI activators are not required for ureteric cell function. The absence of HH signaling in the renal cortex of *WT* mice suggests that the cortex is a zone of low GLI activator and high GLI repressor levels. We determined the importance of exclusion of HH signaling activity from the cortical collecting ducts in mice with *Ptc1*-deficiency targeted to the UB lineage. Absence of ureteric cell *Ptc1*, a negative regulator of the HH signaling pathway, results in ectopic HH signaling activity in the cortical collecting ducts and ureteric bud tips. Ectopic HH signaling activity in the ureteric bud tips, which under normal circumstances is a domain of GLI repressor function, leads to decreased expression of *Ret* and *Wnt11*. These changes result in disruption of ureteric branching morphogenesis and nephrogenesis resulting in renal hypoplasia, likely due to impaired tip function. Remarkably, constitutive GLI3 repressor expression in the *Ptc1^−/−UB^* background, restores ureteric tip cell-specific gene expression and normalizes renal morphogenesis, demonstrating a spatial requirement for GLI3 repressor in the proximal ureteric cells. Together, these results demonstrate a requirement for GLI3 repressor-dependent regulation of nephron number via ureteric tip cell *Wnt11*- and *Ret*-dependent functions.

### Regulation of Ureteric Tip Cell Differentiation by GLI3 Repressor

We have established that loss of GLI3 repressor impairs ureteric tip cells by reducing *Ret* and *Wnt11* expression. The precise mechanism by which GLI-dependent signaling may control *Ret* and *Wnt11* is unclear. Decreased expression of *Wnt11* precedes a decrease in *Ret* expression in the ureteric bud tips of *Ptc1^−/−UB^* kidneys. *Wnt11* maintains *Gdnf* expression in the mesenchyme [20]. Thus, it is probable that reduced *Gdnf* expression in *Ptc1^−/−UB^* mice is secondary to decreased *Wnt11* expression in ureteric tip cells. Consistent with this, *Wnt11* null mice exhibit mild renal hypoplasia and a reduction in *Gdnf* expression [20] almost identical to that observed in *Ptc1^−/−UB^* mutants.

The mosaic expression of *Ptc1-lacZ* expression in the ureteric tips suggests that not all tip cells were exposed to ectopic HH signaling. This explains, in part, why *Ret* and *Wnt11* expression is not completely abolished from the ureteric tips and why expression levels are variable among tips within the same kidney. It is tempting to speculate that the extent of ‘chimerism’ of a ureteric tip may influence the efficiency of its function. The ability of ‘mutant’ tips to confer some function in *Ptc1^−/−UB^* kidneys could account for the mild phenotype since complete abolishment of *Gdnf/Ret* signaling would result in renal agenesis [34], [35], [36]. Impairment of tip cell gene expression has also been reported in mice with deletion of β-catenin targeted to the ureteric cells [37]. Consistent with our findings, β-catenin-deficient mice demonstrate reduced expression of *Ret*, *Wnt11* and *Gdnf* and dilated ureteric tips. However, while early ureteric branching is normal in *Ptc1^−/−UB^* kidneys, it is arrested in β-catenin mutants resulting in severe renal dysplasia. Thus, it is likely that some residual inducing function is maintained in the ureteric tips in *Ptc1^−/−UB^* mice during early stages of kidney development. However, the participation of *Gdnf*, *Ret* and *Wnt11* in an autoregulatory feedback loop to regulate ureteric branching morphogenesis is likely to exacerbate the early decrement in *Wnt11*, *Ret*, and *Gdnf* contributing to the progressively more severe branching phenotype we observed in *Ptc1^−/−UB^* mice.

*Gli2* and *Gli3* can serve both redundant activator and repressor functions in a number of mammalian tissues [31], [38], [39]. Therefore, it is not surprising that the effect of *Gli3*-deficiency alone is less severe than that observed in conditional deletion of *Ptc1*. Constitutive activity of the HH signaling pathway, such as that in *Ptc1^−/−UB^* kidneys, is expected to inhibit the formation of both *Gli2* and *Gli3* repressor isoforms. However, the capacity for the formation of *Gli2* repressor isoforms remains intact in *Gli3*-deficient kidneys allowing for partial redundancy. Early lethality prior to the onset of metanephric development in the majority of *Gli2;Gli3* compound null mutants [31] limits the ability to investigate this functional redundancy in greater detail.

HH signaling is a known regulator of proliferation in mammalian tissues. Indeed, mutations in *Ptc1* are associated with increased incidence of tumorigenesis [12]. In contrast, we observed a moderate decreased in ureteric cell proliferation in *Ptc1*-deficient kidneys. *Gdnf/Ret* signaling has been shown to activate intracellular signaling pathways including the ERK MAP kinase pathway [40]. In the kidney, activation of this pathway leads to cellular events, including cell proliferation, cell survival and migration, and inhibition of this pathway results in reduced ureteric branching [41]. Therefore, reduced *Ret* and *Gdnf* expression in *Ptc1^−/−UB^* kidneys could result in the observed reduction in proliferation. Consistent with this, the absence of a phenotype in *Smo^−/−UB^* kidneys suggests HH signaling is not required for ureteric cell proliferation.

Inactivating mutations in *Gli3* and *Ptc1* have been identified in humans with Greig Cephalopolysyndactyly Syndrome (GCPS) and Nevoid Basal Cell Carcinoma Syndrome (NBCCS, also known as Gorlin Syndrome) respectively [32], [42]. Pertinent to the phenotype, mutations would lead to loss of GLI3 repressor and PTC1-deficiency respectively. Yet, currently no known association between human *Gli3* inactivation or *Ptc1* mutations and renal malformation exists. This is likely due to a number of reasons. Firstly, analysis of kidneys in patients with GCPS or NBCCS has not been performed. Our results provide a basis for analyses of kidney size in affected individuals. Secondly, the degree of haploinsufficiency in humans with *Ptc1* mutations may be insufficient to result in a renal phenotype. *Ptc1* heterozygous mutant mice exhibit phenotypes similar to those observed in humans with BNCCS [12], yet we did not observe any renal abnormalities in these mice. Furthermore, *Ptc1* homozygous null mice are embryonic lethal suggesting that *Ptc1* homozygous null mutations in humans are also incompatible with life [12]. Further, mutational analysis in humans exhibiting sporadic renal hypoplasia for genes involved in HH signaling, including *Gli3* and *Ptc1*, is also warranted, as done for other genes [43], [44].

### SHH Does Not Signal in a Autocrine Manner During Renal Morphogenesis

We show that HH signaling activity is not required in the ureteric bud lineage for normal renal morphogenesis. This is consistent with previous results demonstrating metanephric mesenchyme as the primary target of SHH signaling [11]. Analysis of mice deficient in *Shh* the ureteric bud lineage revealed that *Shh* secreted by the epithelium of the ureter and distal collecting ducts acts on the surrounding mesenchyme to promote cell proliferation and regulate the timing and patterning of smooth muscle progenitor differentiation [11]. Since we have genetically eliminated *Smo* in the ureteric bud only, *Shh* is still capable of paracrine signaling, acting on the surrounding stroma and mesenchyme. In addition, recent *in vitro* and *in vivo* data in the pancreas, has suggested additional, non-canonical, mechanisms of GLI activation, downstream of SMO, via two HH-unrelated pathways, RAS and TGFβ [45], [46], [47]. Whether non-canonical GLI activation occurs during renal morphogenesis remains unknown.

### A Model of Spatial GLI Activator and Repressor Functional Domains

**Figure 6:**
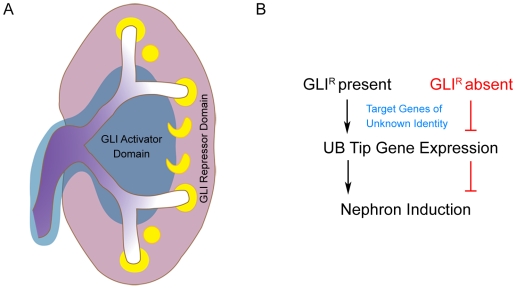
Model for HH signaling function in the ureteric epithelium. (A) During normal renal morphogenesis, *Shh* secreted from the medullary collecting ducts (purple) establishes a gradient of HH signaling resulting in distinct medullary GLI activator (blue) and cortical GLI repressor domains. While HH signaling activity has no functional requirement in the ureteric cell lineage, the absence of HH signal, and thereby function of GLI3 repressor is critical for ureteric tip cell-specific gene expression and subsequent nephron induction (B). In the absence of cortical GLI repressor, such as in *Ptc1^−/−UB^* mice, ureteric tip cell-specific gene expression is impaired resulting in reduced nephrogenesis and renal hypoplasia.

We propose a model whereby distinct SHH-dependent medullary GLI activator domain and cortical repressor domain functions are critical for normal renal ureteric patterning and function (Figure 6). It is likely these domains are established by a SHH gradient, emitted by the ureteric cells of the medullary collecting ducts. While SHH signaling is not required in the medullary collecting ducts themselves, the absence of signal in cortical ureteric cells, contributed to by diminishing SHH concentration and/or pathway inhibitors, is critical for ureteric tip cell gene expression required for ureteric branching and nephron induction. Identification of GLI3 repressor gene targets will provide novel insights into this novel mechanism of ureteric tip cell regulation and function. The presence of renal agenesis/dysplasia in humans with Pallister-Hall syndrome and GLI3 repressor dominant murine models [9], [10] suggests that a fine spatial and lineage-specific balance of GLI activator and GLI repressor must be maintained for normal renal morphogenesis. Furthermore, these results implicate misregulation of HH signaling as a possible underlying mechanism in unexplained human renal dysplasia.

## Materials and Methods

### Ethics Statement

Experiments using mice were approved in advance by the Animal Ethics Committee at The Hospital for Sick Children and were carried out in accordance with the ‘*Canadian Council of Animal Care*.’

### Mice

*Hoxb7creEGFP* mice [17] were mated to *Ptc1^+/−^*, *Ptc1^+/lacZ^* [12] or *Smo^+/−^* [15] mice to generate *Hoxb7creEGFP;Ptc^+/−^*, *Hoxb7creEGFP;Ptc^+/lacZ^* and *Hoxb7creEGFP;Smo^+/−^* males. These males were mated to homozygous *Ptc1* conditional (*Ptc1^neo/neo^*) [48] or *Smo* conditional (*Smo^c/c^*) [49] females to generate *Hoxb7creEGFP;Ptc1^−/neo^*, *Hoxb7creEGFP;Ptc1^lacZ/neo^* and *Hoxb7creEGFP;Smo^−/c^* progeny in which *Ptc1* or *Smo* was specifically removed from the UB lineage. These kidneys are referred to as ‘*Ptc1^−/−UB^*’ or ‘*Smo^−/−UB^*’ in the text. *Hoxb7creEGFP;Ptc1^−/neo^*, *Hoxb7creEGFP;Ptc1^lacZ/neo^* mutant kidneys were indistinguishable, displaying the same phenotype. *Gli3^Δ699/+^* mice [9] were mated to *Ptc1^neo/neo^* mice to ultimately generate *Ptc1^neo/neo^;Gli3^Δ699/+^* mice. *Hoxb7creEGFP;Ptc1^+/−^* or *Hoxb7creEGFP;Ptc1^+/lacZ^* males were mated to *Ptc1^neo/neo^;Gli3^Δ699/+^* females to generate *Hoxb7creEGFP;Ptc1^−/neo^;Gli3^Δ699/+^* or *Hoxb7creEGFP;Ptc1^lacZ/neo^;Gli3^Δ^* ^699/+^ progeny, referred to as ‘*Ptc1^−/−UB^;Gli3^Δ699/+^*’ in the text. *Gli3^XtJ/+^* heterozygote mice [32] were intercrossed to generate *Gli3^XtJ/XtJ^* embryos.

Analysis of HH signaling activity was achieved by mating *Ptc1-lacZ* mice [12] to *Hoxb7creEGFP* mice or *Smo^c/c^* mice to generate *Hoxb7creEGFP;Ptc1^+/lacZ^* males or *Smo^c/c^;Ptc1^+/lacZ^* females. *Hoxb7creEGFP;Ptc1^+/lacZ^* males were mated to *Ptc1^neo/neo^* females to generate *Hoxb7creEGFP;Ptc1^lacZ/neo^* (*Ptc1^lacZ/−UB^*) progeny that contain the *Ptc1-lacZ* reporter allele and in which *Ptc1* is specifically removed from the UB lineage. *Hoxb7creEGFP;Smo^+/−^* males were mated to *Smo^c/c^;Ptc1^+/lacZ^* females to generate *Hoxb7creEGFP;Smo^−/c^;Ptc1^+/lacZ^* progeny that contain the *Ptc1-lacZ* reporter allele and in which *Smo* is eliminated from the UB lineage. PCR genotyping for each allele was performed as previously described [12], [15], [17], [48], [49]. *Gli3^XtJ^* heterozygote and homozygous mice were genotyped according to their characteristic limb phenotypes [32], [50].

Embryonic day 0.5 (E0.5) was considered to be noon on the day of the plug. Littermates were used for all experiments in which normal and mutant embryos were compared.

### β-galactosidase staining

Whole kidneys were briefly fixed in *lacZ* fix solution (25% gluteraldehyde, 100 nM EGTA, 1 M MgCl_2_, 0.1 M sodium phosphate) and rinsed in wash buffer (0.1 M sodium phosphate buffer, 2% nonidet-P40), 1 M MgCl_2_). Kidneys were then placed in *lacZ* staining solution (25 mg/ml X-gal, potassium ferrocyanide, potassium ferricyanide) at 37°C overnight in the dark. Once staining had occurred the reaction was terminated in wash buffer and post-fixed in 10% buffered formalin at 4°C. Whole kidneys were photographed using a Lieca EZ4D dissecting microscope and processed for embedding in paraffin wax and sectioned at 5 µm. Sections were counterstained with eosin or nuclear fast red.

### Histology and immunohistochemistry

Paraffin-embedded kidney sections were analyzed by histology after generating 4 µm tissue sections and staining with haematoxylin and eosin. Immunofluorescence was performed on formalin-fixed, paraffin-embedded kidney sections using anti-Pax2 (Covance, Berkley, CA, 1∶100 dilution), anti-pan cytokeratin (Sigma, St Louis, MO, 1∶100 dilution), *Lotus Tetragonolobus* lectin (LTL) (Vector Laboratories, Burlingame, CA, 1∶100 dilution), anti-NCAM (Sigma, 1∶50 dilution), anti-WT1 (Santa Cruz, Santa Cruz, CA, 1∶500 dilution), anti-α smooth muscle actin (αSMA) (Sigma, 1∶500 dilution), anti-uroplakin III (Progen Biotechnik, Heidelberg, Germany, 1∶10 dilution), anti-Cited1 (Labvision, 1∶500 dilution). Alexa 488 goat anti-mouse and Alexa 568 goat anti-rabbit (Molecular Probes, Carlsbad, CA, 1∶1000 dilution) were used as secondary antibodies. Whole mount immunofluoresence was performed as described [51] with anti-Calbindin-D_28K_ (Sigma, 1∶200 dilution; secondary is Alexa 488 goat anti-mouse, Molecular Probes, 1∶100 dilution) and *Dolichos Biflorus Agglutin* (DBA)-lectin (Vector Laboratories, 1∶200 dilution).

### *In situ* mRNA hybridization

Whole embryos were fixed in 4% PFA in PBS for 16 h at 4°C. *In situ* hybridization was performed on paraffin-embedded sections (4 µm) using DIG-labeled cDNA probes encoding *Ret*, *Wnt11*, *Gdnf*, *Wnt4*, *Six2, Wnt7b, Wnt9b* and *Osr1* as previously described [52].

### *In situ* TUNEL assay, BrdU incorporation assay

Terminal deoxynucleotidyl transferase (TdT)-mediated dUTP nick end labeling (TUNEL) was performed using formalin fixed paraffin-embedded tissue sections as described in the manufacturer's instructions (Promega, Madison, WI). Cell proliferation was assayed in formalin fixed paraffin-embedded kidney tissue by incorporation of 5-bromo-2-deoxyuridine (BrdU, Roche Molecular Biochemicals, Mannheim, Germany), as described [53]. Briefly, pregnant females received an intraperitoneal injection of BrdU (100 mg/g of body weight) 2 h prior to sacrifice. BrdU-positive cells were identified using anti-BrdU peroxidase-conjugated antibody as described (Boehringer, Mannhiem). Immunoreactivity was visualized using Aminoethyl Carbazole horseradish peroxidase chromogen/substrate solution (Zymed Laboratories, USA).

### Ureteric bud isolation and real-time reverse transcriptase-PCR

Kidneys from E11.5 *WT, Smo^−/−UB^* and *Ptc1^lacZ/−UB^* mice were dissected and incubated in DMEM/Hams-F12 culture media (GIBCO) containing 10% FCS and 0.2% Collagenase-B (Roche Diagnostics) for 10 min at 37°C. Kidneys were washed in ice-cold media containing 10% FCS and ureteric buds and metanephric mesenchyme were isolated by microdissection using 30 g needles. Ureteric buds were stored in RNAlater RNA stabilization reagent (Qiagen) and RNA was then isolated using the RNAqueous-Micro RNA Isolation kit (Ambion Inc.). cDNA was generated using First Strand cDNA Synthesis (Invitrogen) from total RNA. Real-time PCR reaction mix contained 1 ng of each cDNA sample, SYBR green PCR Mix (Applied Biosystems) and 300 nM of each primer to a total volume of 25 µl. Primers for *Smo* (Exon 1), *Ptc1* (Exon 3), *Ret* and *Wnt11* were designed using Primer 3 software and verified using the UCSC genome bioinformatics website (genome.ucsc.edu). Real-time PCR Amplification was performed using the Applied Biosystems 7900 HT fast RT-PCR system. Relative levels of mRNA expression were determined using the standard curve method. Individual expression values were normalized by comparison to *β-2 Microglobulin*.

### Calculation of kidney volume and the number of glomeruli

Kidney volume and glomerular number was measured according to Bertram et al. [54] with the following modifications: kidneys embedded in paraffin were exhaustively sectioned at 5 µm, collected at 100 µm intervals and stained with Haematoxylin and Eosin. The area of the tissue section was measured with AxioVision 4.6.3-SP1 (Zeiss) and multiplied by the section thickness. Total kidney volume is the sum of volumes for each section. Glomeruli were identified by the presence of both a podocyte layer and Bowman's capsule.

### Data analysis

Statistical analysis was performed using GraphPad Prism software (Version 3.01) (GraphPad Software Inc., San Diego, CA). Data were analyzed using a Student's *t*-test (two tailed). A probability of less than 0.05 was considered to indicate statistical significance. Values are given as means±SD or SEM.

## Supporting Information

Figure S1 HH signaling activity in developing murine kidney. *Ptc1-lacZ* expression and thereby HH signaling activity at E18.5. (A–C) In *WT* kidneys, *Ptc1-lacZ* is strongly localized cells surrounding the ureter (not shown) and the medullary stroma (ms). No *Ptc1-lacZ* activity is observed in the distal collecting ducts (dc) or any structures of the renal cortex. (D–F) In *Ptc1^−/−UB^* kidneys, in addition to strong localization of *Ptc1-lacZ* to the cells surrounding the ureter and the medullary stroma, *Ptc1-lacZ* is ectopically expressed in the epithelium of the distal collecting ducts, proximal collecting ducts (pc) and in a mosaic pattern in the ureteric bud tips (tip). n = nephrogenic structure.
(2.47 MB TIF)

Figure S2 HH signaling is not required in the ureteric cell lineage. (A–H) Immunofluorescence analysis of newborn *Smo*-deficient kidneys demonstrated no difference in podocyte differentiation (pod) (A,E), normal patterning of the nephrogenic zone (B,F)(green), and comparable densities of proximal tubules (C,G)(green) and collecting ducts (D,H)(red). (I,M) Ureteric branching morphogenesis is comparable between *Smo^−/−UB^* and *WT* littermates at E12.5. (J,K,L,N,O,P) mRNA in situ hybridization demonstrates normal expression of *Ret* and *Wnt11* in the ureteric bud tips (arrowhead) and *Wnt4* in the developing nephrogenic structures, in *Smo^−/−UB^* kidneys at E13.5.
(4.05 MB TIF)

Figure S3 Exencephally in *Ptc1*-deficient mutants. (A–F) Macroscopic analysis of *Ptc1*-deficient mice at all embryonic time points examined demonstrates severe exencephaly (arrowhead), with 100% penetrance. (G) No difference in body weights was detected between *WT* and *Ptc1^−/−UB^* embryos (*WT* vs. *Ptc1^−/−UB^*: 1.49+0.13 vs. 1.57+0.13, p>0.05). (H) Quantitative real-time PCR of E11.5 isolated ureteric buds. *Ptc1* mRNA transcripts are increased 50-fold in *Ptc1^−/−UB^* ureteric cells (*WT* vs. *Ptc1^−/−UB^*: 2.23+0.2 vs. 108.51+6.47, p<0.001).
(1.26 MB TIF)

Figure S4 Quantitation of *Ptc1*-deficient kidneys. (A,B) No histological differences were observed between *WT* and *Ptc1^−/−UB^* kidneys at E13.5. (C,D) NCAM (red) positive nephrogenic structures where similar between *WT* and *Ptc1^−/−UB^* kidneys. (E) Quantitation of nephrogenesis at E13.5 demonstrates no significant difference in the number of NCAM positive nephrogenic structures in *WT* and *Ptc1*-deficient kidneys. (F) Quantitation of ureteric branching morphogenesis at E12.5 demonstrates no significant difference in branch number in *WT* and *Ptc1^−/−UB^* kidneys. n  =  nephrogenic intermediate structure, ub  =  ureteric epithelium.
(0.79 MB TIF)

Figure S5 *Ptc1*-deficiency does not effect metanephric cell survival. (A–D) Analysis of apoptosis in E13.5 kidney tissue using the TUNEL. TUNEL-positive cells (brown color) are rarely detected in the ureteric bud (ub) of *WT* or *Ptc1^−/−UB^* kidneys. There is no observable difference in TUNEL-positive cells in the mesenchyme (mes) between *WT* and *Ptc1*-deficient kidneys. (C,D) Quantitative analysis of ureteric bud and mesenchymal apoptosis. (C) Ureteric cell apoptosis, quantitated as the percent of TUNEL-positive ureteric cells, was not significantly altered in *Ptc1*-deficient kidneys (*WT* vs. *Ptc1^−/−UB^*: 0.32+0.13 vs 0.41+0.28, p = 0.78). (D) Mesenchymal cell apoptosis, quantitated as the number of TUNEL-positive cells per mm^2^ of renal tissue was comparable in *WT* and *Ptc1^−/−UB^* kidneys (*WT* vs. *Ptc1^−/−UB^*: 0.23+0.03 vs 0.19+0.04, p = 0.46). n  =  nephrogenic structure. (E–H) Whole mount Calbinidin-D_28K_ and DBA-lectin immunofluorescence at E13.5. Calbindin-D_28K_ is expressed in both ureteric stalks and tips in *WT* and *Ptc1^−/−UB^* kidneys (E,F). DBA-lectin localizes predominantly to the ureteric stalk in *WT* kidneys and is excluded from the ureteric tips (G). In *Ptc1^−/−UB^* kidneys DBA-lectin is observed throughout the ureteric tips and ureteric stalks (H). (I,J) *Wnt7b* is expressed in the ureteric stalks (arrow) but is absent from ureteric tips (arrowhead) in both *WT* and *Ptc1^−/−UB^* kidneys.
(2.29 MB TIF)

Figure S6 *Ptc1*-deficiency does not effect the nephron progenitor population. (A,D) RNA *in situ* hybridization demonstrates normal expression of *Ors1* and *Six2* in the mesenchymal precursor population of *Ptc1*-deficient kidneys. (E,F) Cited-1 immunofluorescence is comparable between *WT* and *Ptc1^−/−UB^* kidneys. (G,H) RNA *in situ* hybridization demonstrates a reduced number of developing nephrogenic structures in *Ptc1^−/−UB^* kidneys but those present exhibit normal expression of *Wnt4*. (I,J) *Wnt9b* expression is comparable between *WT* and *Ptc1^−/−UB^* kidneys.
(3.66 MB TIF)

Figure S7 *Ptc1*-deficiency does not effect differentiation of the distal epithelium. (A–F) Uroplakin immunofluorescence (red color, arrows) demonstrates normal differentiation of the urothelium in the renal pelvis of *Ptc1*-deficient kidneys at E18.5. (C,F) Ectopic uroplakin-positive epithelium was not observed in the proximal epithelium. (G–L) Immunofluorescence for αSMA (red color, arrowheads) demonstrates normal smooth muscle differentiation surrounding the ureter and renal pelvis in *Ptc1*-deficient kidneys. (I,L) Ectopic smooth muscle differentiation was not observed surrounding the proximal epithelium. rp = renal pelvis.
(3.26 MB TIF)

Figure S8 Effect of *Gli3* inactivation of metanephric differentiation.(A–L) Immunofluorescence analysis of metanephric differentiation markers. *Gli3*-deficient kidneys demonstrated no difference in podocyte differentiation (aqua) (A,B), normal patterning of the nephrogenic zone (C,D)(green), comparable densities of proximal tubules (E,F)(green), comparable densities of collecting ducts (G,H)(red) and normal differentiation of smooth muscle (red) (I,J) and urothelium (red) (K,L).
(3.80 MB TIF)

Figure S9 Early metanephric development in *Gli3^Delta699/+^* mice is normal. RNA *in situ* hybridization demonstrates normal expression of *Ret*, *Wnt11* and *Gdnf* in *Gli3^Delta699/+^* kidneys at E13.5.
(1.03 MB TIF)

Table S1 Mutant Mouse Frequency
(0.04 MB DOC)

Table S2 Mutant Mouse Frequency
(0.04 MB DOC)
