# Supplementary figures and images for: GLI3 Repressor Controls Nephron Number via Regulation of Wnt11 and Ret in Ureteric Tip Cells
Source: PLoS One. 2009 Oct 7;4(10):e7313. doi: 10.1371/journal.pone.0007313 (PMC2754339; doi:10.1371/journal.pone.0007313)

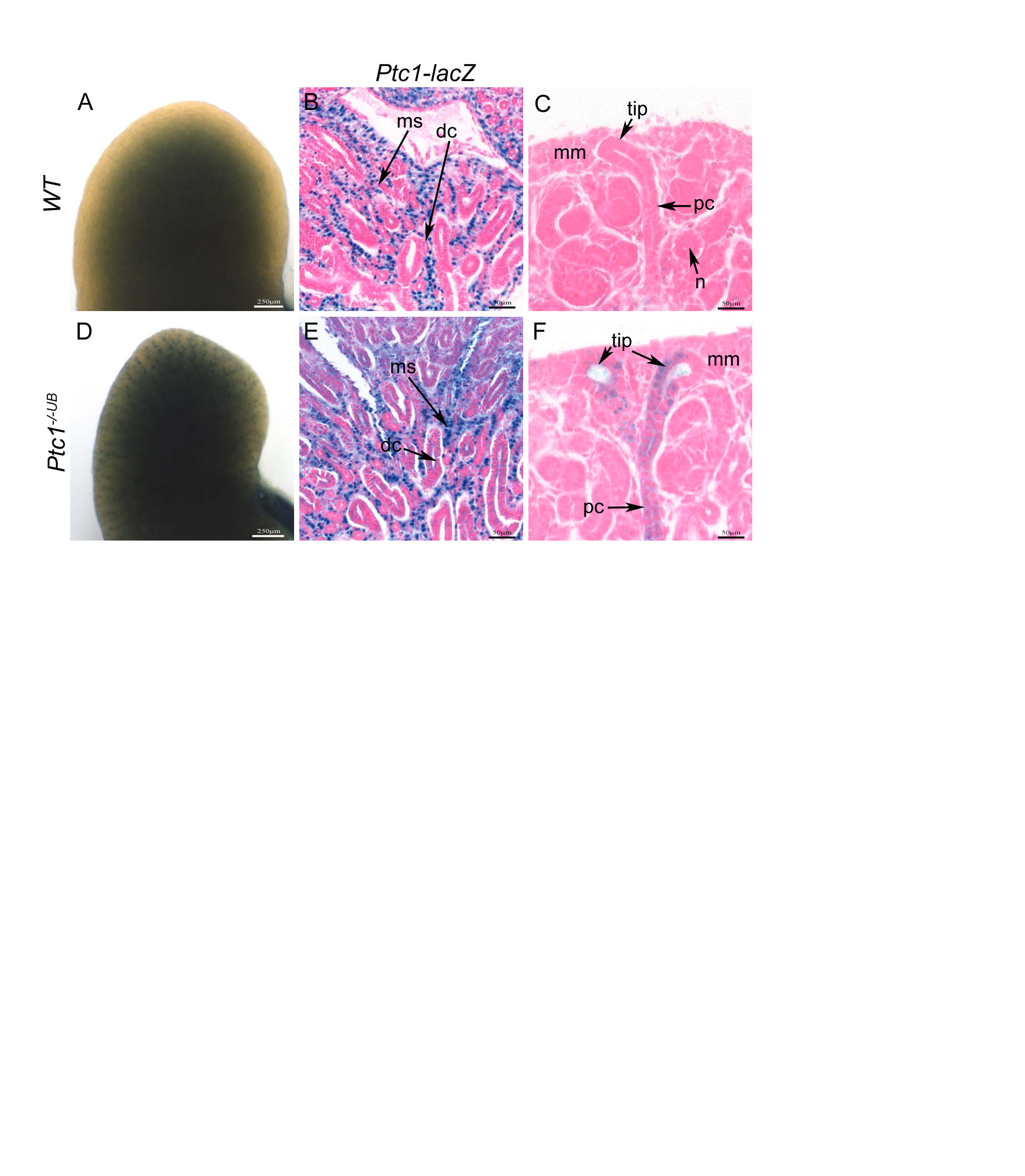

Supplement: Figure S1 — HH signaling activity in developing murine kidney. Ptc1-lacZ expression and thereby HH signaling activity at E18.5. (A–C) In WT kidneys, Ptc1-lacZ is strongly localized cells surrounding the ureter (not shown) and the medullary stroma (ms). No Ptc1-lacZ activity is observed in the distal collecting ducts (dc) or any structures of the renal cortex. (D–F) In Ptc1−/−UB kidneys, in addition to strong localization of Ptc1-lacZ to the cells surrounding the ureter and the medullary stroma, Ptc1-lacZ is ectopically expressed in the epithelium of the distal collecting ducts, proximal collecting ducts (pc) and in a mosaic pattern in the ureteric bud tips (tip). n = nephrogenic structure. (2.47 MB TIF) [file pone.0007313.s001.tif]

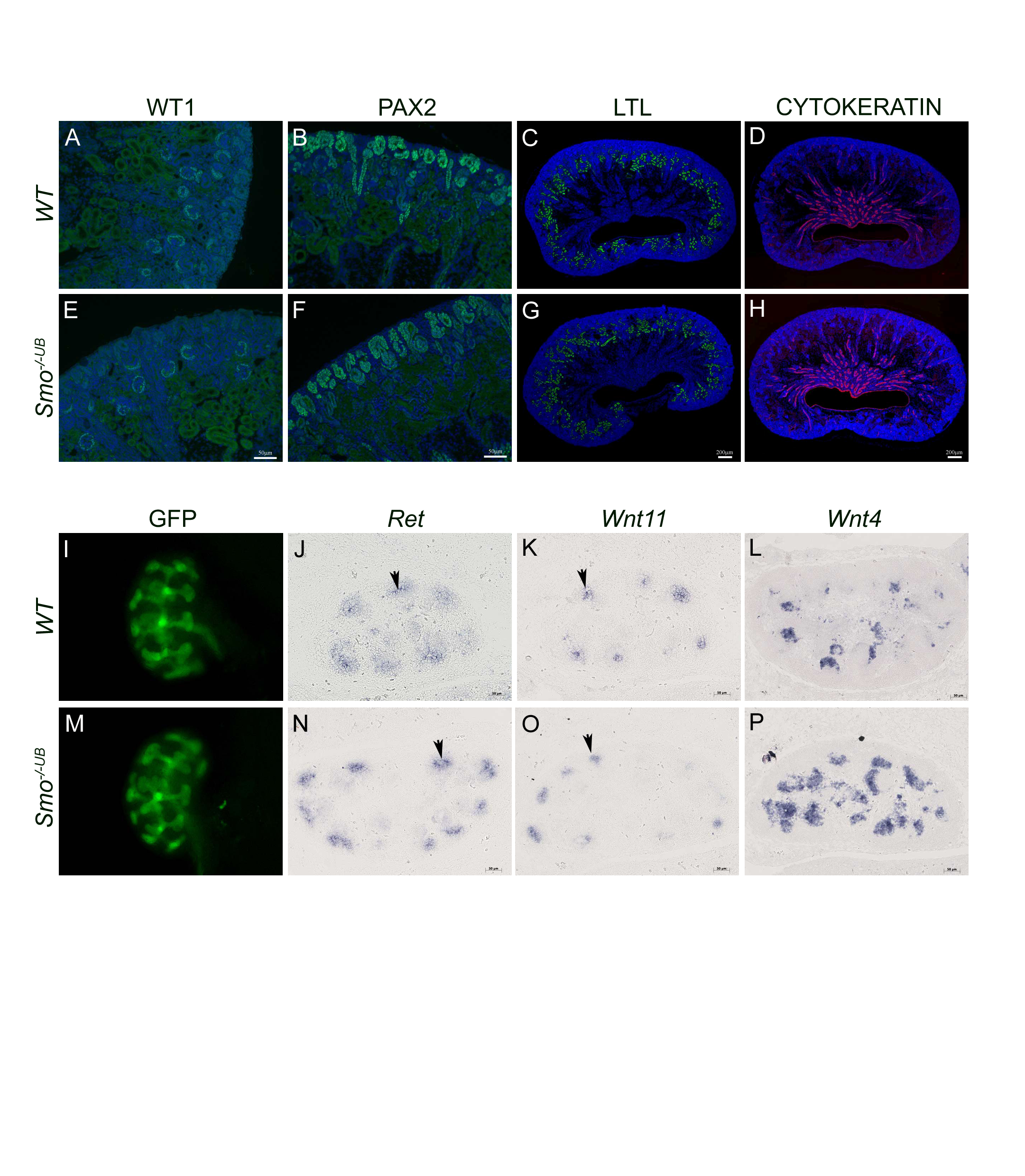

Supplement: Figure S2 — HH signaling is not required in the ureteric cell lineage. (A–H) Immunofluorescence analysis of newborn Smo-deficient kidneys demonstrated no difference in podocyte differentiation (pod) (A,E), normal patterning of the nephrogenic zone (B,F)(green), and comparable densities of proximal tubules (C,G)(green) and collecting ducts (D,H)(red). (I,M) Ureteric branching morphogenesis is comparable between Smo−/−UB and WT littermates at E12.5. (J,K,L,N,O,P) mRNA in situ hybridization demonstrates normal expression of Ret and Wnt11 in the ureteric bud tips (arrowhead) and Wnt4 in the developing nephrogenic structures, in Smo−/−UB kidneys at E13.5. (4.05 MB TIF) [file pone.0007313.s002.tif]

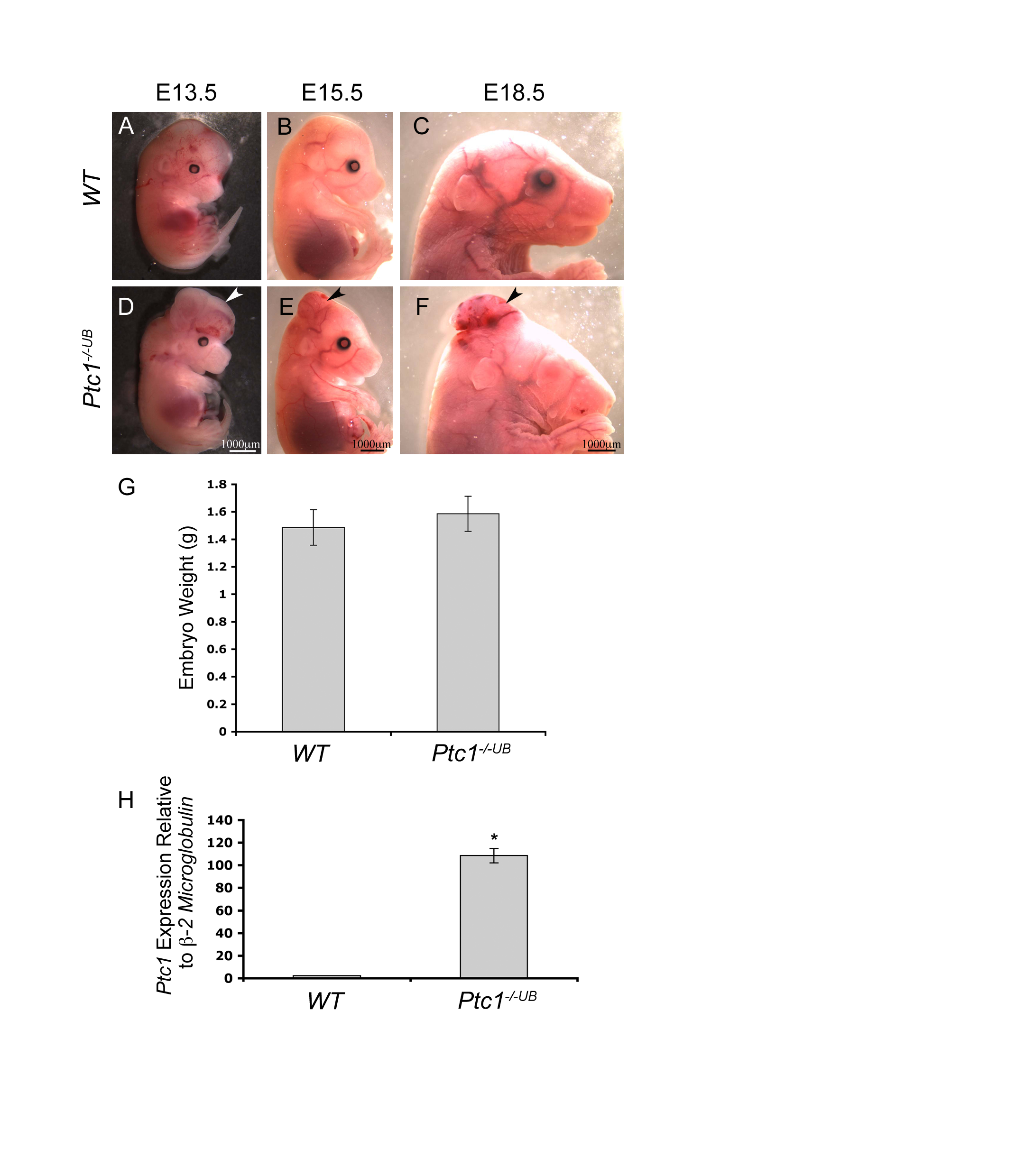

Supplement: Figure S3 — Exencephally in Ptc1-deficient mutants. (A–F) Macroscopic analysis of Ptc1-deficient mice at all embryonic time points examined demonstrates severe exencephaly (arrowhead), with 100% penetrance. (G) No difference in body weights was detected between WT and Ptc1−/−UB embryos (WT vs. Ptc1−/−UB: 1.49+0.13 vs. 1.57+0.13, p>0.05). (H) Quantitative real-time PCR of E11.5 isolated ureteric buds. Ptc1 mRNA transcripts are increased 50-fold in Ptc1−/−UB ureteric cells (WT vs. Ptc1−/−UB: 2.23+0.2 vs. 108.51+6.47, p<0.001). (1.26 MB TIF) [file pone.0007313.s003.tif]

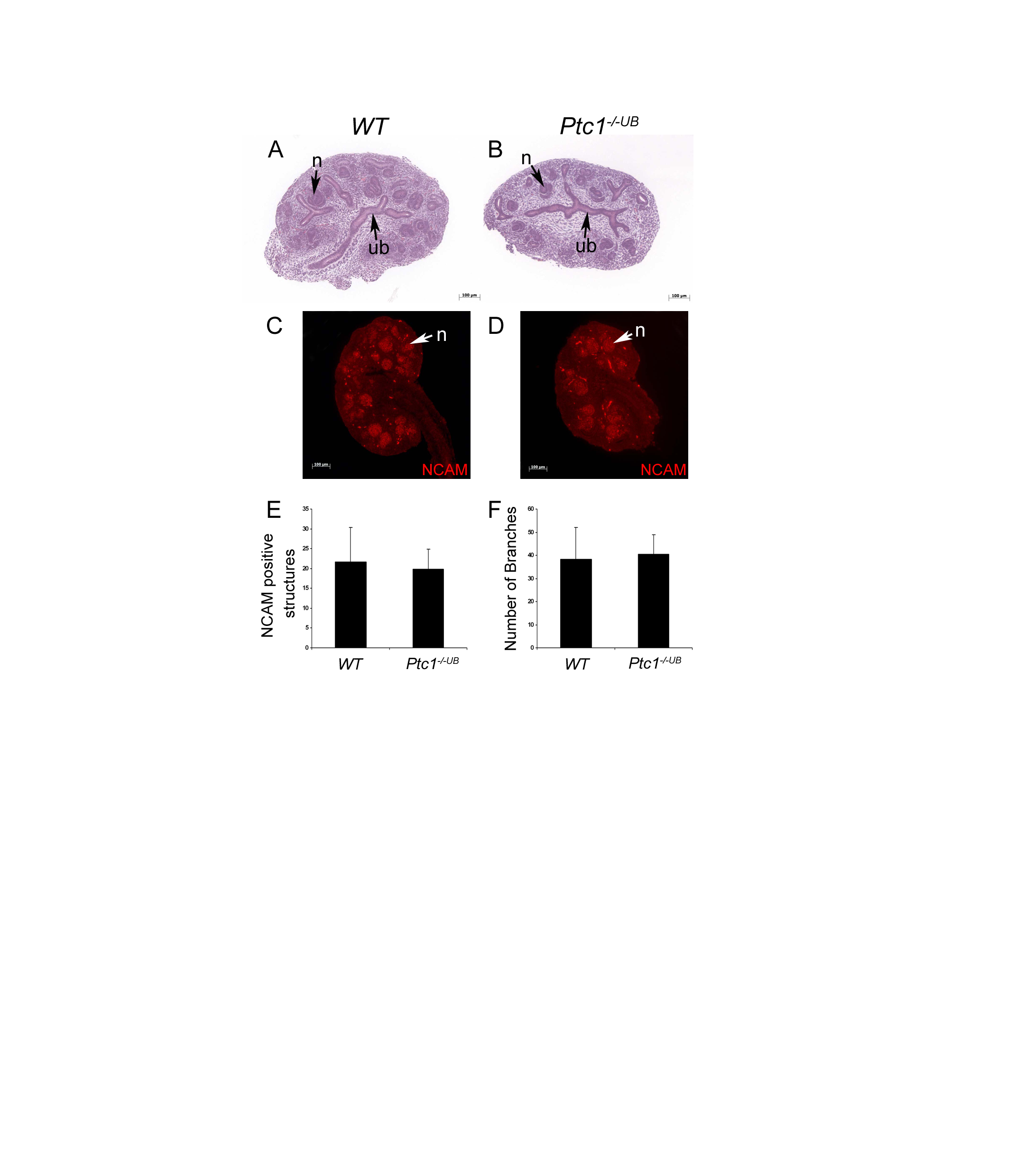

Supplement: Figure S4 — Quantitation of Ptc1-deficient kidneys. (A,B) No histological differences were observed between WT and Ptc1−/−UB kidneys at E13.5. (C,D) NCAM (red) positive nephrogenic structures where similar between WT and Ptc1−/−UB kidneys. (E) Quantitation of nephrogenesis at E13.5 demonstrates no significant difference in the number of NCAM positive nephrogenic structures in WT and Ptc1-deficient kidneys. (F) Quantitation of ureteric branching morphogenesis at E12.5 demonstrates no significant difference in branch number in WT and Ptc1−/−UB kidneys. n = nephrogenic intermediate structure, ub = ureteric epithelium. (0.79 MB TIF) [file pone.0007313.s004.tif]

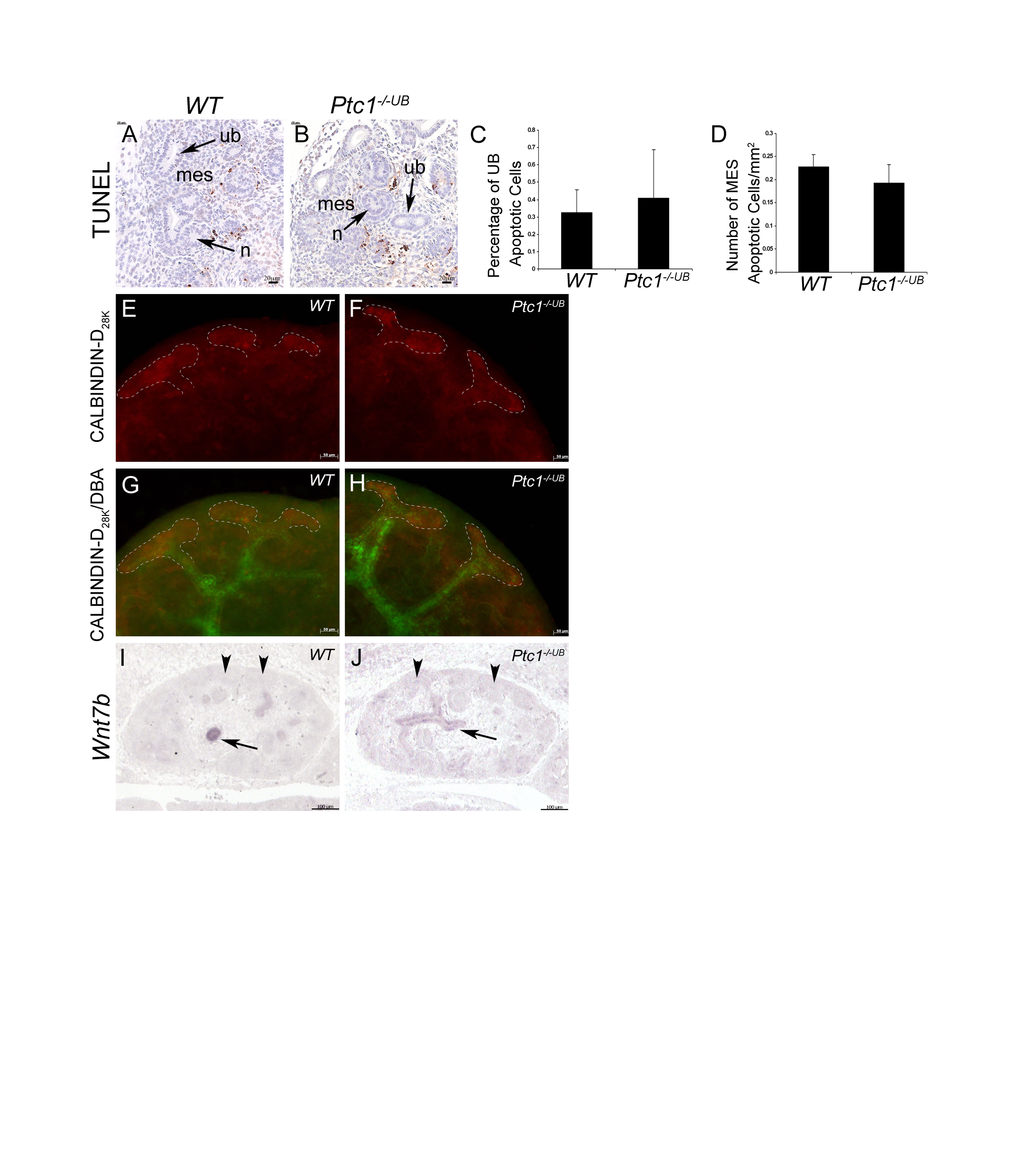

Supplement: Figure S5 — Ptc1-deficiency does not effect metanephric cell survival. (A–D) Analysis of apoptosis in E13.5 kidney tissue using the TUNEL. TUNEL-positive cells (brown color) are rarely detected in the ureteric bud (ub) of WT or Ptc1−/−UB kidneys. There is no observable difference in TUNEL-positive cells in the mesenchyme (mes) between WT and Ptc1-deficient kidneys. (C,D) Quantitative analysis of ureteric bud and mesenchymal apoptosis. (C) Ureteric cell apoptosis, quantitated as the percent of TUNEL-positive ureteric cells, was not significantly altered in Ptc1-deficient kidneys (WT vs. Ptc1−/−UB: 0.32+0.13 vs 0.41+0.28, p = 0.78). (D) Mesenchymal cell apoptosis, quantitated as the number of TUNEL-positive cells per mm2 of renal tissue was comparable in WT and Ptc1−/−UB kidneys (WT vs. Ptc1−/−UB: 0.23+0.03 vs 0.19+0.04, p = 0.46). n = nephrogenic structure. (E–H) Whole mount Calbinidin-D28K and DBA-lectin immunofluorescence at E13.5. Calbindin-D28K is expressed in both ureteric stalks and tips in WT and Ptc1−/−UB kidneys (E,F). DBA-lectin localizes predominantly to the ureteric stalk in WT kidneys and is excluded from the ureteric tips (G). In Ptc1−/−UB kidneys DBA-lectin is observed throughout the ureteric tips and ureteric stalks (H). (I,J) Wnt7b is expressed in the ureteric stalks (arrow) but is absent from ureteric tips (arrowhead) in both WT and Ptc1−/−UB kidneys. (2.29 MB TIF) [file pone.0007313.s005.tif]

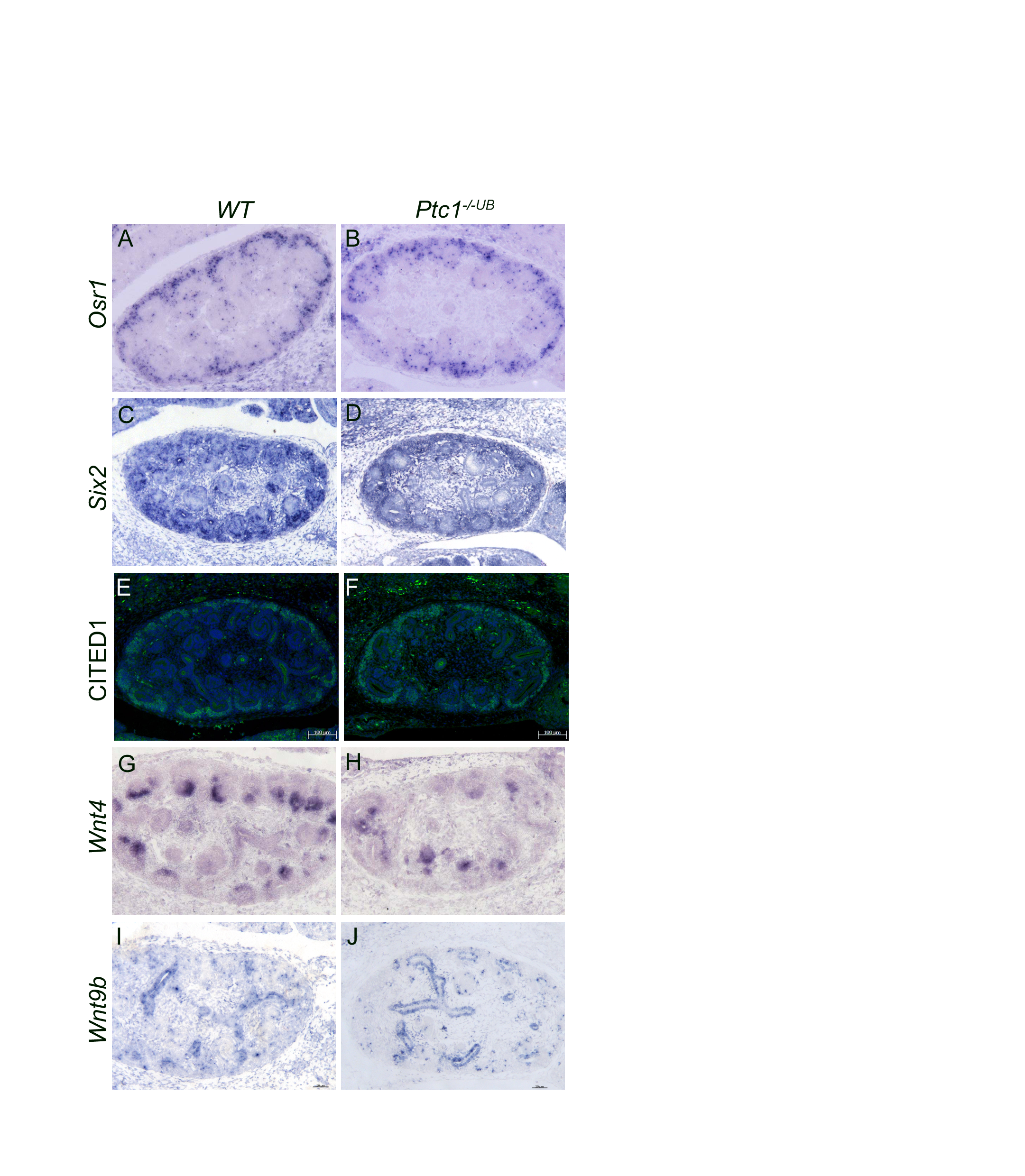

Supplement: Figure S6 — Ptc1-deficiency does not effect the nephron progenitor population. (A,D) RNA in situ hybridization demonstrates normal expression of Ors1 and Six2 in the mesenchymal precursor population of Ptc1-deficient kidneys. (E,F) Cited-1 immunofluorescence is comparable between WT and Ptc1−/−UB kidneys. (G,H) RNA in situ hybridization demonstrates a reduced number of developing nephrogenic structures in Ptc1−/−UB kidneys but those present exhibit normal expression of Wnt4. (I,J) Wnt9b expression is comparable between WT and Ptc1−/−UB kidneys. (3.66 MB TIF) [file pone.0007313.s006.tif]

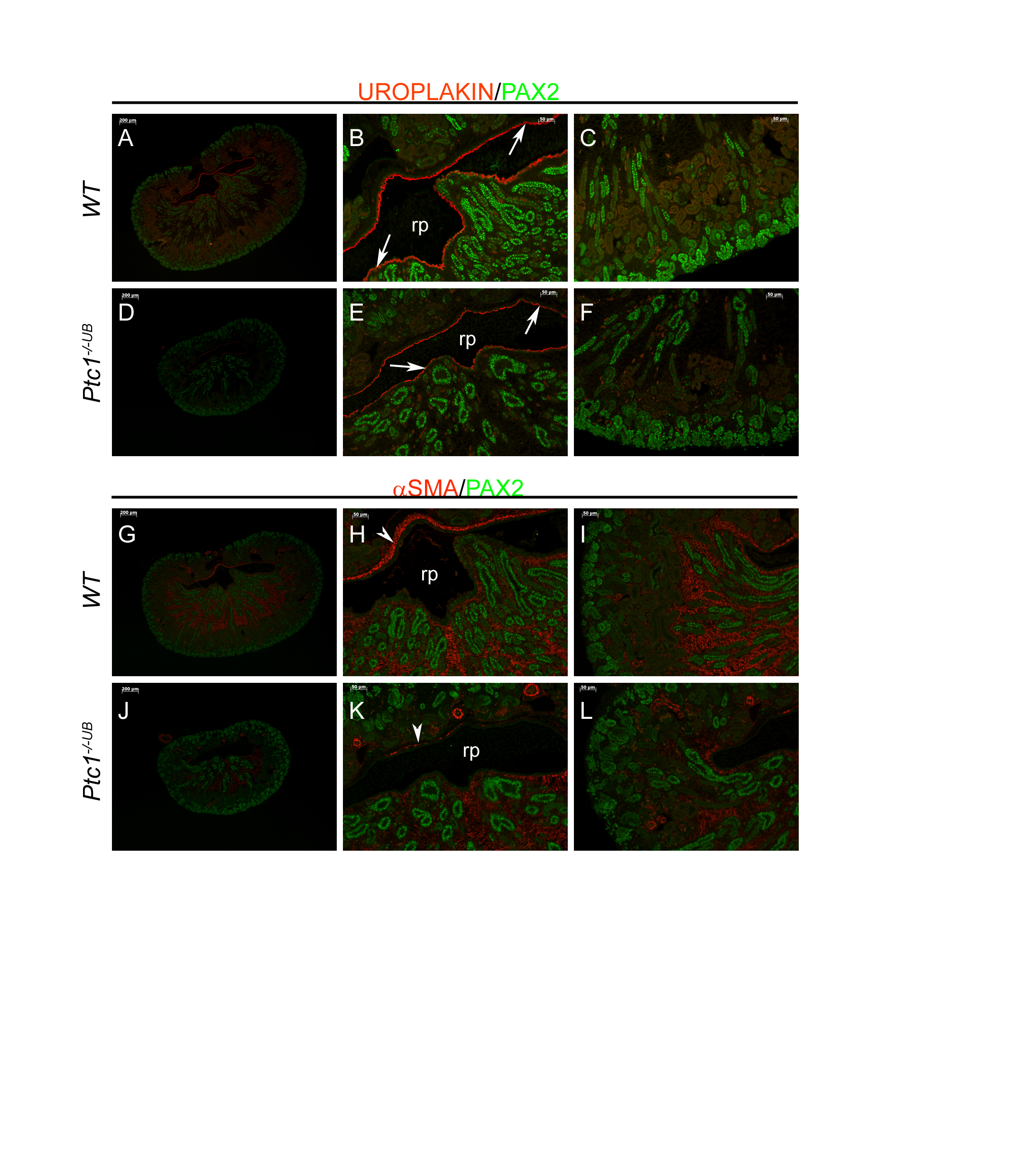

Supplement: Figure S7 — Ptc1-deficiency does not effect differentiation of the distal epithelium. (A–F) Uroplakin immunofluorescence (red color, arrows) demonstrates normal differentiation of the urothelium in the renal pelvis of Ptc1-deficient kidneys at E18.5. (C,F) Ectopic uroplakin-positive epithelium was not observed in the proximal epithelium. (G–L) Immunofluorescence for αSMA (red color, arrowheads) demonstrates normal smooth muscle differentiation surrounding the ureter and renal pelvis in Ptc1-deficient kidneys. (I,L) Ectopic smooth muscle differentiation was not observed surrounding the proximal epithelium. rp = renal pelvis. (3.26 MB TIF) [file pone.0007313.s007.tif]

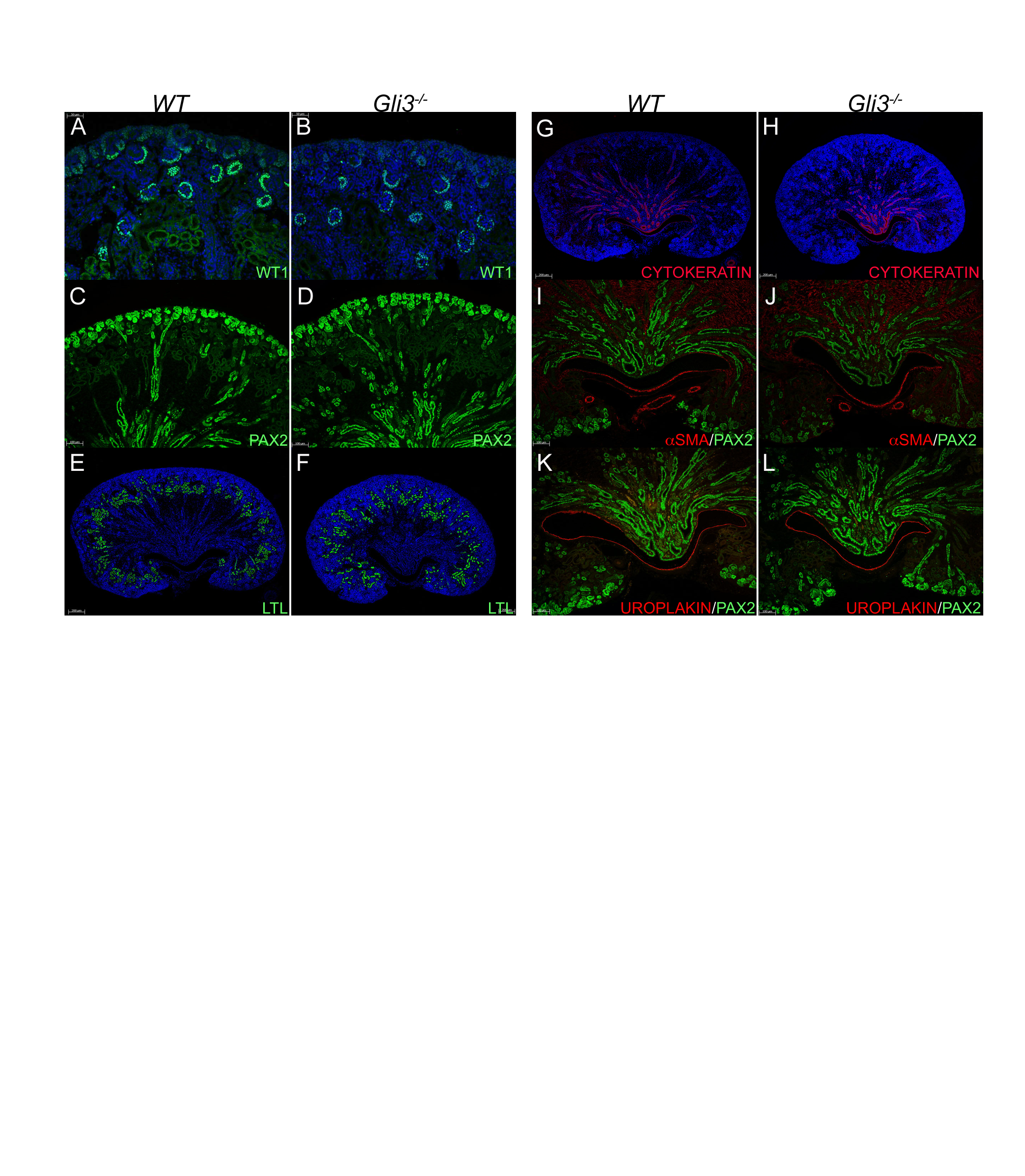

Supplement: Figure S8 — Effect of Gli3 inactivation of metanephric differentiation.(A–L) Immunofluorescence analysis of metanephric differentiation markers. Gli3-deficient kidneys demonstrated no difference in podocyte differentiation (aqua) (A,B), normal patterning of the nephrogenic zone (C,D)(green), comparable densities of proximal tubules (E,F)(green), comparable densities of collecting ducts (G,H)(red) and normal differentiation of smooth muscle (red) (I,J) and urothelium (red) (K,L). (3.80 MB TIF) [file pone.0007313.s008.tif]

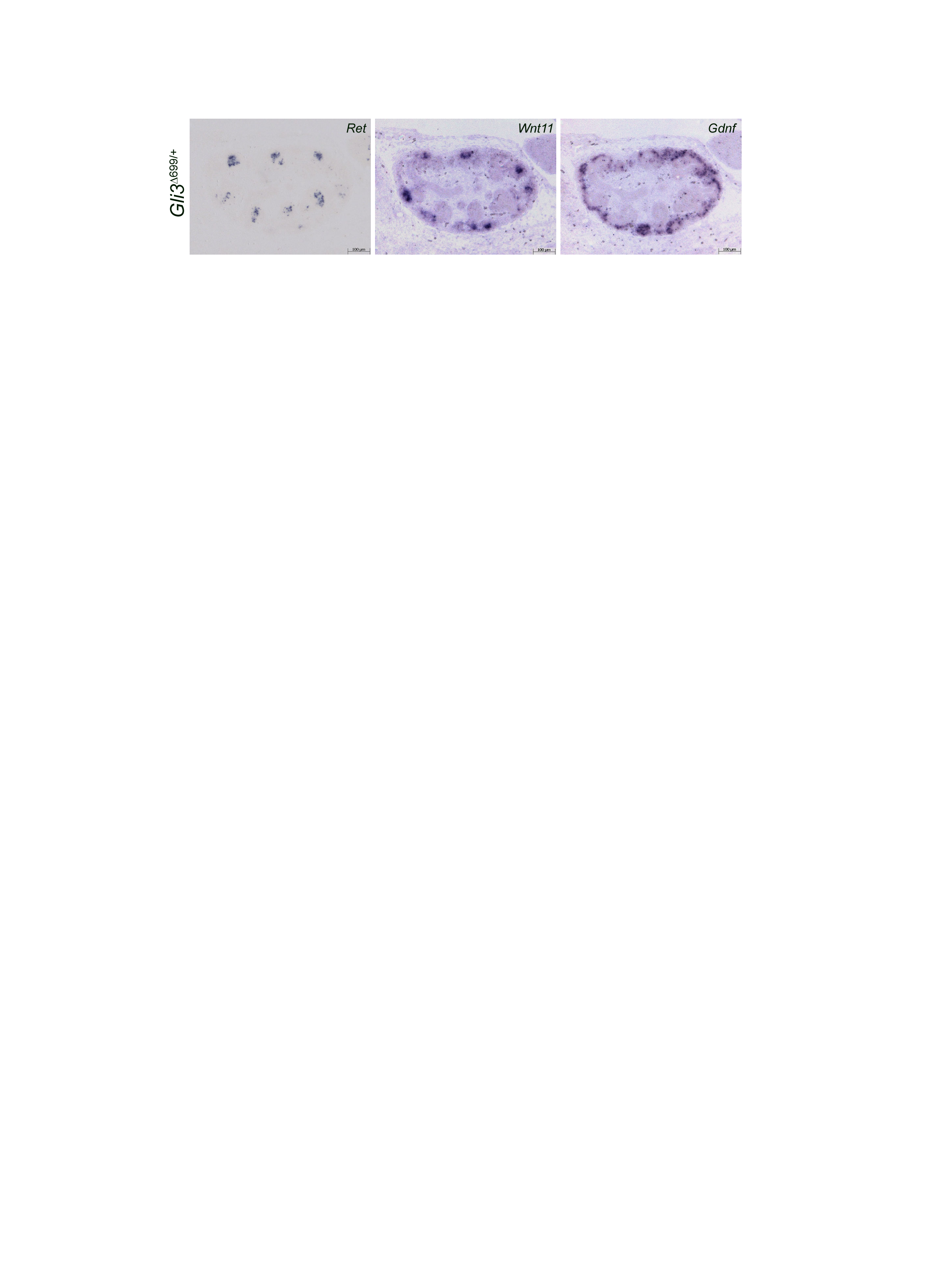

Supplement: Figure S9 — Early metanephric development in Gli3Delta699/+ mice is normal. RNA in situ hybridization demonstrates normal expression of Ret, Wnt11 and Gdnf in Gli3Delta699/+ kidneys at E13.5. (1.03 MB TIF) [file pone.0007313.s009.tif]
