# Supplementary material for: GLI3 Repressor Controls Nephron Number via Regulation of Wnt11 and Ret in Ureteric Tip Cells
Source: PLoS One. 2009 Oct 7;4(10):e7313. doi: 10.1371/journal.pone.0007313 (PMC2754339; doi:10.1371/journal.pone.0007313)
Supplement: Table S1 — Mutant Mouse Frequency (0.04 MB DOC) [file pone.0007313.s010.doc]

Table S1

Mutant Mouse Frequency

|  | ***Smo^+/loxp^*** | ***Smo^-/loxp^*** | ***Smo^+/-UB^*** | ***Smo^-/-UB^*** |
| --- | --- | --- | --- | --- |
|  |  |  |  |  |
| **PN30** | 23.5% (14/60) | 23.5% (14/60) | 25% (15/60) | 28% (17/60) |
| **Newborn** | 20% (17/84) | 31% (26/84) | 30% (25/84) | 19% (16/84) |
|  |  |  |  |  |
| **E18.5** | 41% (7/17) | 18% (3/17) | 29% (5/17) | 12% (2/17) |
| **E15.5** | 10% (2/20) | 35% (7/20) | 20% (4/20) | 35% (7/20) |
| **E13.5** | 29% (12/42) | 21% (9/42) | 29% (12/42) | 21% (9/42) |
| **E12.5** | 22.3% (2/9) | 22.3% (2/9) | 33% (3/9) | 22.3% (2/9) |
|  |  |  |  |  |
| **Total** | 23% (54/232) | 26% (61/232) | 28% (64/232) | 23% (53/232) |
|  |  |  |  |  |
| Genotype | *Smo^+loxp^* | *Smo^-/loxp^* | *Hoxb7Cre;Smo^+/loxp^* | *Hoxb7Cre;Smo^-loxp^* |

Mendelian genetics predict that 25% of littermates from *Hoxb7Cre;Smo^+/-^* x *Smo^loxp/loxp^* crosses will be mutants*.* Viable neonatal *Smo* deficient mutants were recovered in expected Mednelidan ratios at all time points analyzed.
