# Supplementary material for: GLI3 Repressor Controls Nephron Number via Regulation of Wnt11 and Ret in Ureteric Tip Cells
Source: PLoS One. 2009 Oct 7;4(10):e7313. doi: 10.1371/journal.pone.0007313 (PMC2754339; doi:10.1371/journal.pone.0007313)
Supplement: Table S2 — Mutant Mouse Frequency (0.04 MB DOC) [file pone.0007313.s011.doc]

Table S2

Mutant Mouse Frequency

|  | ***Ptc1^+/neo^*** | ***Ptc^-/neo^*** | ***Ptc1^+/-UB^*** | ***Ptc1^-/-UB^*** |
| --- | --- | --- | --- | --- |
|  |  |  |  |  |
| **Newborn** | 27% (16/59) | 35.5% (21/59) | 35.5% (21/59) | 2% (1/59)* |
|  |  |  |  |  |
| **E18.5** | 16% (9/56) | 29% (16/56) | 23% (13/56) | 32% (18/56) |
| **E15.5** | 36% (15/42) | 24% (10/42) | 19% (8/42) | 21% (9/42) |
| **E13.5** | 30% (24/79) | 27% (21/79) | 23% (18/39) | 20% (16/79) |
| **E12.5** | 24% (16/63) | 24% (15/63) | 29% (18/63) | 22% (14/63) |
|  |  |  |  |  |
| **Total Embryonic** | 26% (64/240) | 26% (62/240) | 24% (57/240) | 24% (57/240) |
|  |  |  |  |  |
| Genotype | *Ptc1^+/neo^* | *Ptc^-/neo^* | *Hoxb7Cre;Ptc1^+/neo^* | *Hoxb7Cre;Ptc1^-/-neo^* |

* Dead and partially cannibalized pup.

Mendelian genetics predict that 25% of littermates from *Hoxb7Cre;Ptc1^+/-^* x *Ptc1^neo/neo^* crosses will be mutants*.* Viable neonatal *Ptc1* deficient pups could not be recovered. However, live *Ptc1^-/-UB^* mutant embryos were recovered in expected Mendelian ratios at all embryonic time points analyzed.
